# Quantitative surface field analysis: learning causal models to predict ligand binding affinity and pose

**DOI:** 10.1007/s10822-018-0126-x

**Published:** 2018-06-22

**Authors:** Ann E. Cleves, Ajay N. Jain

**Affiliations:** 1 Helen Diller Family Comprehensive Cancer Center, University of California, San Francisco, USA; 2 Department of Bioengineering and Therapeutic Sciences, University of California, San Francisco, USA

**Keywords:** QSAR, Binding affinity, Machine learning, Multiple-instance learning, Free-energy perturbation, Pose prediction, Confidence estimation

## Abstract

We introduce the QuanSA method for inducing physically meaningful field-based models of ligand binding pockets based on structure-activity data alone. The method is closely related to the QMOD approach, substituting a learned scoring field for a pocket constructed of molecular fragments. The problem of mutual ligand alignment is addressed in a general way, and optimal model parameters and ligand poses are identified through multiple-instance machine learning. We provide algorithmic details along with performance results on sixteen structure-activity data sets covering many pharmaceutically relevant targets. In particular, we show how models initially induced from small data sets can extrapolatively identify potent new ligands with novel underlying scaffolds with very high specificity. Further, we show that combining predictions from QuanSA models with those from physics-based simulation approaches is synergistic. QuanSA predictions yield binding affinities, explicit estimates of ligand strain, associated ligand pose families, and estimates of structural novelty and confidence. The method is applicable for fine-grained lead optimization as well as potent new lead identification.

## Introduction

Binding affinity prediction continues to be a challenge for computer-aided drug design, especially in the case where there is no high-resolution experimental structure of the target of interest. Experimental structure determination remains challenging for many pharmaceutical targets such as ligand-gated ion channels, membrane transporters, and (now to a lesser extent) membrane-spanning G-protein coupled receptors. The most widely used methods for activity prediction in such cases include field-based approaches such as CoMFA (and variants such as CoMSIA, Topomer CoMFA, and CMF) [1–4], pharmacophoric approaches (e.g. Catalyst and Phase) [5–9], and descriptor-based approaches [10, 11]. These methods vary in the degree to which they parallel what is known physically about protein-ligand interactions.

A key attraction of simulation oriented physics-based methods, such as MM/PBSA or MM/GBSA [12–15] or free-energy perturbation (FEP) [16–18], is that, in principle, these approaches are congruent with what we know physically. For the former methods, in the case where experimental structures are known for all ligands under consideration, performance can be quite variable based on the specific protein target (Pearson’s r$^2$ range of 0.0–0.8 for individual proteins) [19], though careful work has shown more consistent results in some cases (r$^2$ of 0.5–0.6 for three enzyme targets) [14]. For the FEP approach, affinity predictions are made by estimating the difference in the free energy of protein-ligand complexes between closely-related ligand pairs (typically just a few substituent atoms are different). Recent advances in force-fields, sampling methods, and automated design of perturbation graphs [18] have yielded results that offer guidance in fine-grained molecular optimization. For single perturbations of a few atoms, errors in predicting changes in free energy were approximately 0.9 kcal/mol.

**Fig. 1:**
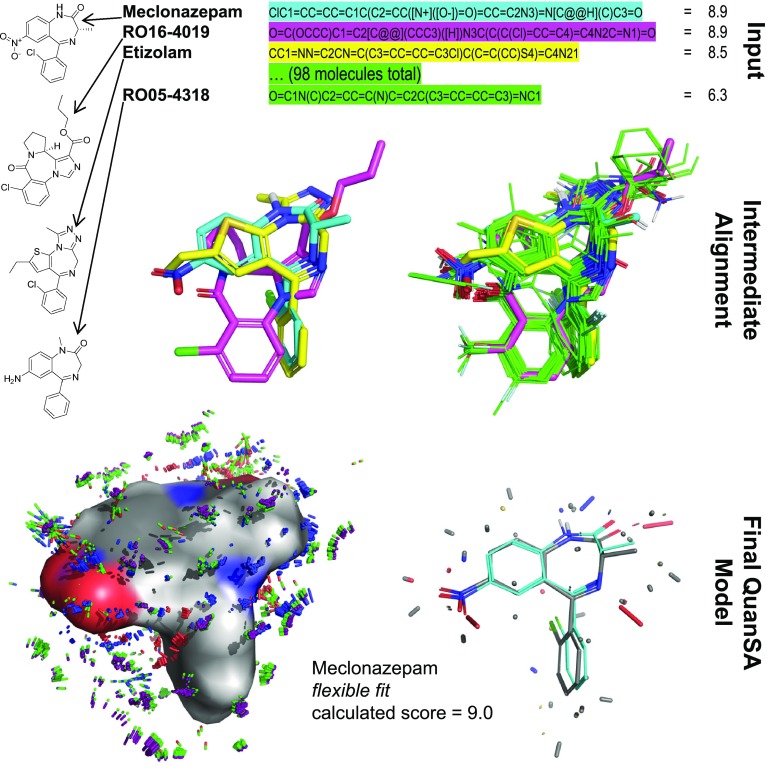
Model induction is fully automatic, beginning with pure structure-activity data (top, SMILES and pK$_i$), generation of core alignments for diverse active ligands and elaboration into full pose cliques (middle, single poses shown without variations), and derivation of a final pocket-field model (lower left), which exhibits adaptation of training ligand poses to the induced model based on optimizing interactions with the model (lower right, initial (cyan) and final (gray) poses of meclonazepam are shown).

QSAR approaches are not restricted to cases where experimentally determined structures are available; they can be applied purely from structure and activity data. However, model construction based on correlative considerations rather than causative ones may explain the frequent disappointment in real-world accuracy of QSAR predictions [20]. We believe that improvements in predictive power for 3D-QSAR methods will follow if methods directly address key physical phenomena that are observed in protein-ligand interactions (see [21] for a more detailed discussion): 1) choice of ligand poses must be defined by the model; 2) the effects of substituent changes on a central scaffold may not be additive [22]; 3) changes in ligand structures induce changes in ligand pose relative to a binding pocket; and 4) molecular activity may depend upon detailed shape complementarity between pocket and ligand.

We introduce a new machine-learning method for induction of models from structure-activity data: *Quan*titative *S*urface-field *A*nalysis (QuanSA). It is related to the QMOD approach [23–27], but rather than constructing a pocket composed of molecular fragments, the method constructs a “pocket-field” that is still physical in nature. Figure 1 illustrates model induction using the benzodiazepine binding site (BZR) of the GABA$_A$ receptor as an example. Beginning with pure SAR data (here SMILES strings and associated pK$_i$ measurements), low-energy conformational ensembles are produced, from which multiple mutual ligand alignments are automatically constructed. Each such alignment contains a single optimal pose for each training ligand along with many related poses.

**Fig. 2:**
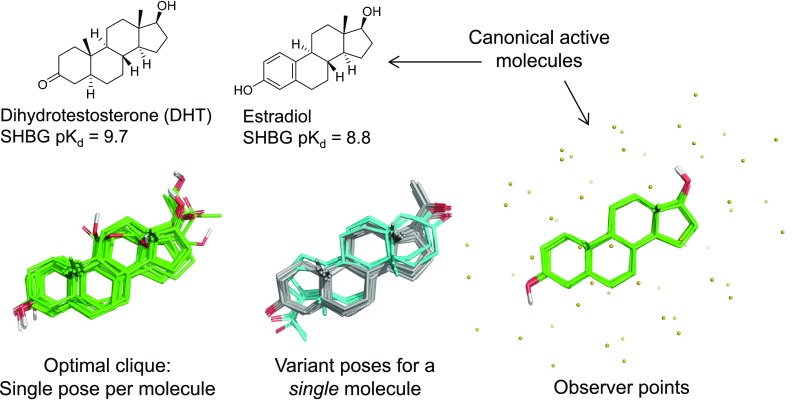
Canonical molecules from the globulin binding sets (top); optimal clique of single poses for each molecule (bottom left); all variants for a *single* molecule (bottom middle); and the observer points shown in relation to the most active SHBG molecules (bottom right).

**Fig. 3:**
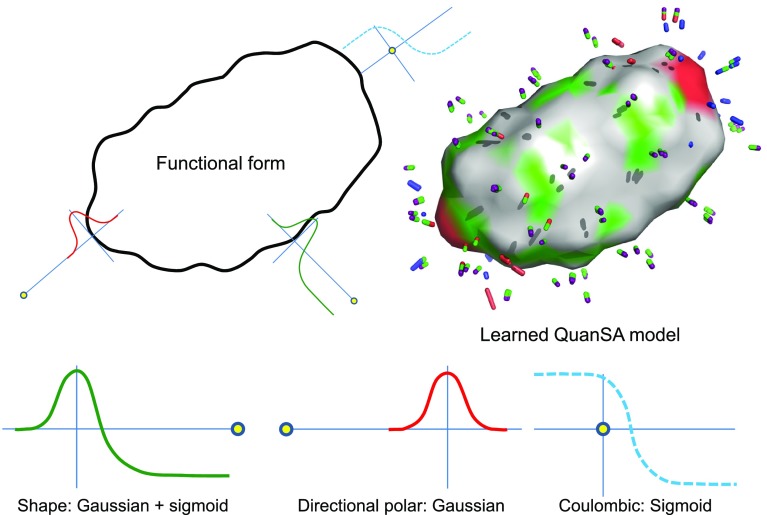
The response functions for QuanSA are computed from observer points (yellow circles); the functions are responsive to molecular shape, hydrogen bond donor/acceptor arrangement (including directionality); and electrostatic field.

The final model (lower left of Fig. 1) has the property that each training ligand in its *optimal* pose (that which maximizes its score within the model) has a score that is close to the experimental activity. The parameters of the pocket-field are depicted by the numerous thin colored sticks in the lower left of Fig. 1 (described in detailed later). These parameters define the shapes of response functions located at observer points in a fixed reference frame around the molecules (shown as yellow spheres and circles in Figs. 2 and 3).

The colored sticks at the bottom right of Fig. 1 indicate the magnitude of the scores attributable to the local portions of the molecule from the observers in that area, with the overall ligand score coming from a sum across the response functions. The score takes molecular strain into account directly, reflecting the degree to which a ligand’s predicted pose deviates from the identified global minimum (using a variant of MMFF94s, implemented within the ForceGen conformer elaboration approach [28]). The final optimal pose of a ligand is typically close to, but not identical to, one of the initial poses from the original alignment process (the final pose in gray carbons compared to the initial pose in cyan carbons) at the bottom right of Fig. 1).

**Fig. 4:**
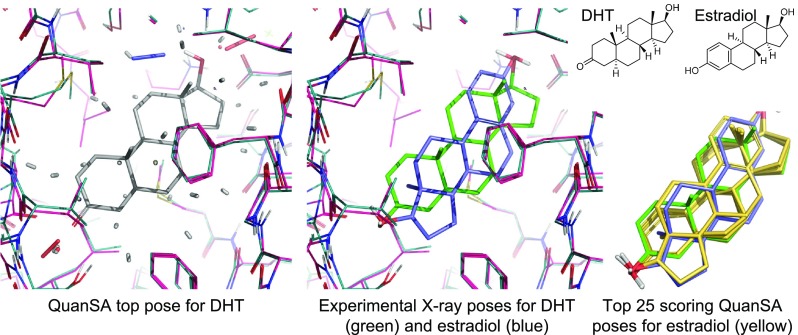
Comparison of pocket-field interactions for the top predicted ligand pose of DHT (left, steric interactions in gray sticks, ligand acceptor to protein donor in red sticks, ligand donor to protein acceptor interactions in blue sticks, and Coulombic interactions in half gray and half red/blue/yellow sticks); experimentally determined ligand poses to the SHBG binding pocket (middle, PDB Codes 1D2S and 1LHU); and QuanSA predicted alternative poses for estradiol (right).

Figure 2 shows two canonical molecules from the steroid QSAR benchmark introduced by Cramer in the original CoMFA paper [1] (the carrier protein previously termed testosterone binding globulin (TBG) is now termed sex-hormone binding globulin (SHBG)). The twenty training molecules are shown in the single highest-scoring clique of one pose per molecule (lower left), all of which make a non-controversial superimposition of the steroid core structure. However, as seen among the variant poses of one molecule (lower middle, cyan and gray carbons), an alternative orientation, flipped both vertically and horizontally, is also produced by the similarity-based alignment procedure. As pointed out by Cherkasov et al. [29], depending on the particular steroid ligand, both orientations have been observed crystallographically in bound complexes with SHBG.

Figure 3 illustrates the functional form of the induced field. At each of the observer points (yellow spheres from Fig. 2), the minimum distance to the molecular surface is calculated, as is the distance and directional congruence to the nearest hydrogen bond donor and acceptor (precisely as in the morphological similarity method [30]). Additionally, the electrostatic potential energy of a fixed-magnitude point charge moved from infinity to the observer position is calculated. So, at each feature point, there are six calculated values: one which measures pure shape by minimum surface distance; two for each donor/acceptor ligand atomic surface which measure distance and directionality; and one responsive to local ligand charge.

The response functions are specific to each type of measured value, and each are parameterized according to the SAR data. The combination Gaussian/sigmoid is computed from the pure shape measurement, with the minimum surface distance at a particular observer point corresponding to the location along the learned curve. The directional polar measurements feed into strictly Gaussian terms, with the distance driving the location within the Gaussian and the directionality modulating the height, with the preferred location and maximal height being learned from the data. The Coulombic local ligand charge measurement feeds into the learned sigmoidal curve.

The sum over all observers of non-linear functions of these values forms the pocket-field score. The parameters of these functions are estimated during the learning process. As with the QMOD and Compass approaches [31–33], model refinement proceeds iteratively along with pose refinement with respect to the evolving model.

Figure 4 shows the relationship between the induced QuanSA pocket-field and the SHBG binding pocket. At left, we see the top-scoring pose for DHT along with the interactions made with the pocket-field. There is good correspondence between the hydrophobic interactions (gray sticks) and the pocket formed by hydrophobic residues such as phenylalanine, methionine, and valine. The precise geometry and direction of the polar interactions differ somewhat from the crystal structure, most obviously at the upper right, where the orientation of the hydroxyl on DHT is flipped relative to what one would expect from the presence of the carboxylate in the pocket. In the middle panel, we see that the orientation of estradiol is flipped from DHT in its bound pose, determined experimentally. While the top-scoring predicted pose of estradiol lines up the steroid core with that of DHT, the set of 25 top-scoring poses includes both orientations. The highest scoring “reversed” pose of estradiol was just 1.3 log units (in terms of pK$_d$) lower than the best pose (shown in the rightmost panel).

We present details of the QuanSA algorithms and results on multiple data sets, including two with particular significance from a QSAR benchmarking perspective, eight from a validation report for free-energy perturbation, four from a recent benchmark where QuanSA pocket-fields were also applied to extensive ChEMBL data, and one case of particular pharmaceutical interest where model refinement was explored. Results were consistently better than those from prior reports on the same benchmarks using other QSAR approaches. A particularly interesting aspect of the physics-based benchmark was that QuanSA performance was quantitatively very close, but the errors between the methods were uncorrelated, so the combined predictions were synergistic with respect to accuracy.

As the difficulty of predictions increased (measured by the similarity of predicted molecules to those available for training) so did deviations from experimentally determined activities. For the easiest cases, where differences of just a few atoms distinguished the test and training molecules, mean absolute error (MAE) was roughly 0.5 pK$_d$ units (0.7 kcal/mol). For challenging cases by conventional QSAR standards, typical errors on blind test molecules were about 0.7 log units (0.9 kcal/mol). In cases where it was possible to test QuanSA models on large sets of ChEMBL data, typical errors were higher, ranging from 0.9–1.5 log units (1.2–2.0 kcal/mol), but the models were able to identify potent and structurally novel molecules with high specificity and produced highly statistically significant rank orderings of potency.

QuanSA is implemented as a new module within the Surflex Platform, first released with Version 4.2.

## Methods, data, and computational protocols

The QuanSA approach is built upon the morphological similarity method that underlies Surflex-Sim [30] and also upon the QMOD method, particularly benefiting from more recent refinements [27]. Here, we will describe the algorithmic details, followed by the molecular data sets, and finally the computational procedures.

### QuanSA response functions

Recall that the QMOD approach constructs a binding site model by choosing a set of small molecular fragments (“probes”) and refining their positions (a “pocketmol”). The procedure is carried out such that the intermolecular docking score between a ligand and the pocketmol [34], when optimized for both alignment and conformation, will be close to the experimentally determined activity (units of pK${_d}$). The physical effect of the probes that make up the pocketmol is made through the docking scoring function, whose positive interactions are principally composed of Gaussian steric and polar terms (the latter with a directional aspect) and whose negative interactions are dominated by a quadratic interpenetration penalty [35] as well as weaker sigmoidal and Gaussian penalties (for interpenetration and polar repulsion, respectively). So, the pocketmol, while being represented as molecular fragments, is actually producing a functional scoring field whose behavior is controlled by the precise composition of the pocketmol.

However, in the QMOD approach, a molecular fragment is either present or absent, with no weighting. This limits the possibility for representing certain aspects of protein flexibility. For example, a valine sidechain may have multiple orientations of very similar energy, where a ligand can make a favorable contact in one case using a small substituent but clash with the protein using a larger substituent unless the valine assumes a different orientation. In such a case, placement of a probe to mimic the position of the valine that is favorable to the small ligand substituent *automatically* precludes the larger one. Similarly, there are many cases where a protein hydroxyl may adopt multiple orientations, making it possible for a ligand to favorably present either a donor or an acceptor in very similar positions.

**Fig. 5:**
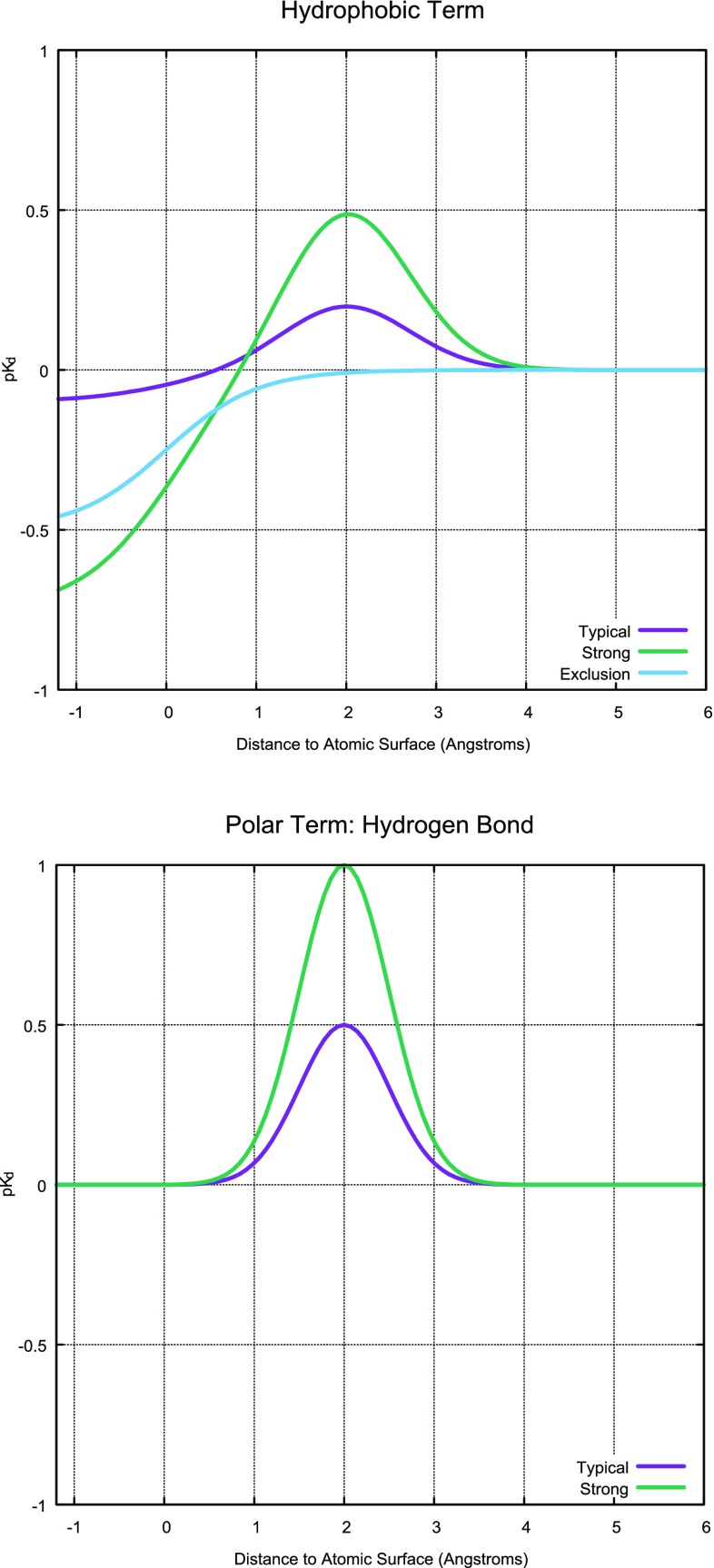
Typical response functions for steric and polar terms are very similar in effect to the scoring function of Surflex-Dock.

Situations such as these present problems for QMOD, and this is fundamentally an issue of *inductive bias* in the method. Within the QMOD formalism, because it makes use of molecular fragments to model pocket interactions, the presence of a favorable steric interaction carries with it an assumption about its magnitude and a necessary hard constraint in interpenetration (these values come from the Surflex-Dock scoring function). Similarly, the presence of a favorable polar interaction with a ligand donor carries with it the assumption that a ligand acceptor would be unfavorable. For the QuanSA approach, it was possible to make different choices regarding the inductive bias that is represented by the functional response terms so that they would be more congruent with flexible protein binding pockets.

#### Steric shape and hydrogen-bond terms

The QuanSA approach is capable of representing the interaction types that QMOD constructs, but it is also capable of representing interactions that can easily capture protein pocket movement. As seen from Fig. 3, the response functions of QuanSA are Gaussian and sigmoidal in nature, following Eqs. 1 and 2:

**Fig. 6:**
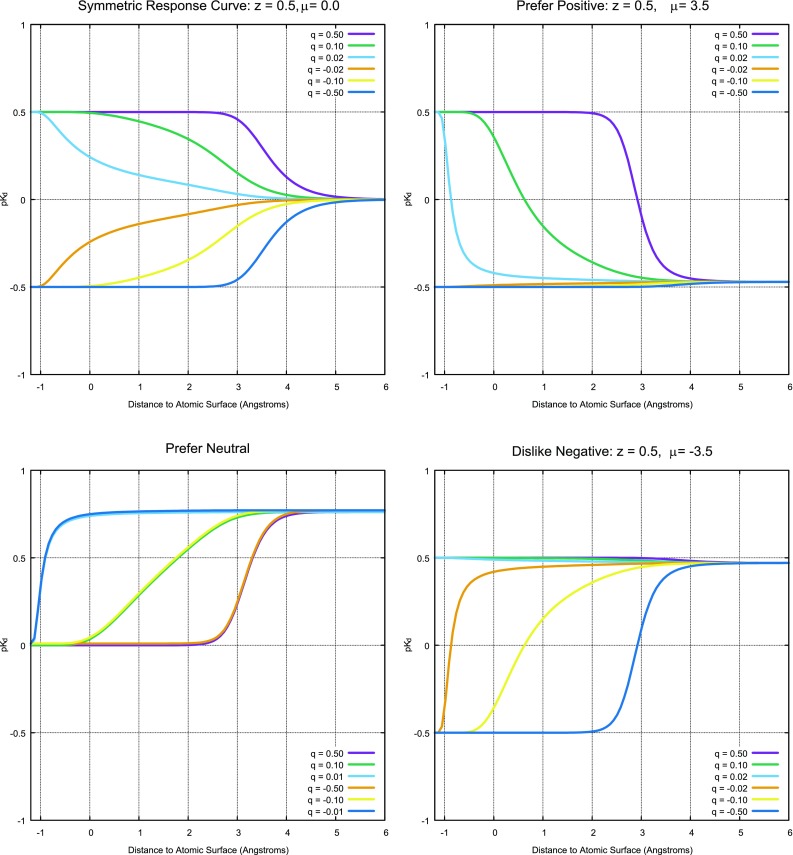
The response functions for the electric field of the ligand can be quite complex, offering the ability to learn common physical interactions in protein-ligand complexes that include protein movement and the presence of complex entropic and water effects.

1 $$\begin{aligned} g(x,\sigma )&= e^{-x^2/\sigma } \end{aligned}$$

2 $$\begin{aligned} s(x,\alpha )&= \frac{1}{1+e^{-x/\alpha }} \end{aligned}$$

For a ligand in a given pose ($L_p$), at a particular observer point *i*, given the minimum distance to the molecular surface $r_i$, the hydrophobic/steric term is governed by the three learned parameters $z^S_i$, $\mu ^S_i$, and $p^S_i$:

3 $$\begin{aligned} d_i&= r_i - \mu ^S_i \end{aligned}$$

4 $$\begin{aligned} S_i&= z^S_i \cdot g(d_i,c_1) - p^S_i \cdot s((d_i+c_2),c_3) \end{aligned}$$

here $c_1 = 1$ (the width of the Gaussian), $c_2 = 2$ (affecting the degree of interpenetration allowed without penalty), and $c_3 = 1/2$ (the steepness of the sigmoidal term). The three learned parameters are constrained to be non-negative for this term, which can also be thought of as a shape term. Note that all of the response terms are dependent on the ligand pose, but $L_p$ is omitted for clarity.

At a particular observer point *i*, given the minimum distance to a hydrogen-bond donor, denoted $r^D_i$, the corresponding term is governed by the learned parameters $z^D_i$, and $\mu ^D_i$ (with $c_4 = 1/2$, affecting the width of the Gaussian):

5 $$\begin{aligned} D_i&= z^D_i \cdot g(r^D_i - \mu ^D_i,c_4) \times M^D_i \end{aligned}$$

The two learned parameters are constrained to be non-negative for the hydrogen-bonding terms. The $M^D_i$ value (directional magnitude) is determined by the directional correspondence between the donor or acceptor and the observer point location, possibly scaled upward by formal charge, as detailed previously in the definition of the morphological similarity function [30]. The hydrogen-bond acceptor term *A* is defined analogously:

6 $$\begin{aligned} A_i&= z^A_i \cdot g(r^A_i - \mu ^A_i,c_4) \times M^A_i \end{aligned}$$

Figure 5 depicts typical examples of learned shape and hydrogen-bond donor terms, shown with ideal surface distances from the observer point of 2.0Å. In the case of the shape term, the favorable interaction magnitude can vary from zero to typically less than 0.5 pK$_d$ units. The unfavorable interaction magnitude, which occurs at closer than ideal distances between observer point and molecular surface, can be zero but its typical range is larger than for the magnitude of the Gaussian portion. In sharp contrast to the QMOD approach, it is possible to learn subtle differences in these magnitudes as well as to model a “wall” in a pocket that carries no favorable interaction possibilities (blue line). These different qualitative types of response functions (and varying magnitudes) are all possible within protein binding pockets when considering flexibility and the energetic costs of movement.

Similarly, for the hydrogen-bond interaction terms, QuanSA can learn differing magnitudes for the value of H-bond interactions as well as either/or behavior from the perspective of a single observer point. Typical ranges for the *z* values of these terms are 0.0–1.0, as seen in Fig. 5. The directional aspect of the calculation is important. If, for example, a donor proton is oriented such that the X-H–Observer angle is 120$^{\circ }$ rather than the ideal 180$^{\circ }$ the strength of the interaction is reduced by half.

#### Coulombic effects

A sharp departure from the QMOD approach is the direct measurement of electric field effects from the estimated partial charges at the atomic centers of the ligand (the charges are estimated as described in [28], closely related to the electronegativity equalization approach introduced by Gilson [36]). At each observer point, the potential energy of a point charge of 0.2e (moved from an infinite distance to the observer’s location) is calculated, with r$_{ij}$ denoting the distance from observer *i* to atom *j* and the partial charge of atom *j* being $q_j$, as follows:

7 $$\begin{aligned} \epsilon (x)&= \frac{1}{1 - s(x+c_5,c_6)} \end{aligned}$$

8 $$\begin{aligned} E_i&= \sum \limits _{j=1}^{n} \frac{q_jc_7}{\epsilon (r_{ij})(r_{ij}+\delta )} \end{aligned}$$

The units of $E_i$ are in kcal/mol (with $c_7 = 332.0716$). The definition of the dielectric, with $c_5 = 4$ and $c_6 = 1/2$, produces a value of 1 at close distances, and it begins to increase at a distance of approximately one water shell, then it increases without bound becoming effectively infinite at interatomic center distances of 6.0Å or more. By defining the Coulombic feature values $E_i$ in this manner, they measure local electrostatic effects primarily, and they have a computationally convenient cutoff that allows skipping of ligand atoms above a threshold distance. The Coulombic response terms ($C_i$) are defined as functions of $E_i$, controlled by two learned parameters per observer point, $z^C_i$ and $\mu ^C_i$:

9 $$\begin{aligned} C_i&= z^C_i \cdot (s(E_i-\mu ^C_i,c_8) - 1/2) \end{aligned}$$

The $\mu ^C_i$ values can be thought of as charge preference inflection points. With $\mu ^C_i = 0$ and $z^C_i> 0$, when $E_i$ is positive, then $C_i$ will be as well. As $\mu ^C_i$ increases, a larger positive value of $E_i$ is required to produce a positive value of $C_i$. Here, $c_8 = 1.0$, which produces a gentle slope of response to changes in the electrostatic field.

Neither of the two learned Coulombic parameters are constrained as to sign, and the learned response curves can represent numerous different cases, as illustrated in Fig. 6. The upper left plot shows the simplest case conceptually, where a positive charge moving near an observer point is favorable (purple, green, and light blue curves), with larger effects at further distances as charge magnitude increases. The converse is true for negative charges. This corresponds to the situation where a formal negative charge sits at the bottom of a hydrophobic well, for example in a trypsin-like serine protease S1 pocket.

At the top right of Fig. 6, we see the case where a positive charge is preferred, but where the difference between a neutral or negative charge is minimal. Such cases can occur when, for example, a mobile protein carboxylate can shift to make a favorable interaction with a positive ligand charge in a particular location, but where the ligand remains well-solvated if the ligand has insufficient positive charge in that location. A related case can occur where a negative charge is *unfavorable* but neutral or positive are equally acceptable (bottom right). By flipping the sign of $z^C_i$, the converse response curves result: symmetric preference for negative charge, “prefer negative,” and “dislike positive.”

A hydrophobic concavity will tend to trap water molecules, and displacement of the water can be quite favorable in terms of free energy. This displacement is not easily modeled using Coulombic electrostatics (the magnitudes of the charges on the ideal ligand are small). This case can be learned by the QuanSA approach by two adjacent observer points, where one learns “dislike positive” and the other learns “dislike negative” (shown at bottom left of Fig. 6). In such a case, small local charges of either polarity are preferred in proximity to the observer point pair (light and dark blue lines), with increasing unfavorability as charge increases (green/yellow and orange/purple curves).

**Fig. 7:**
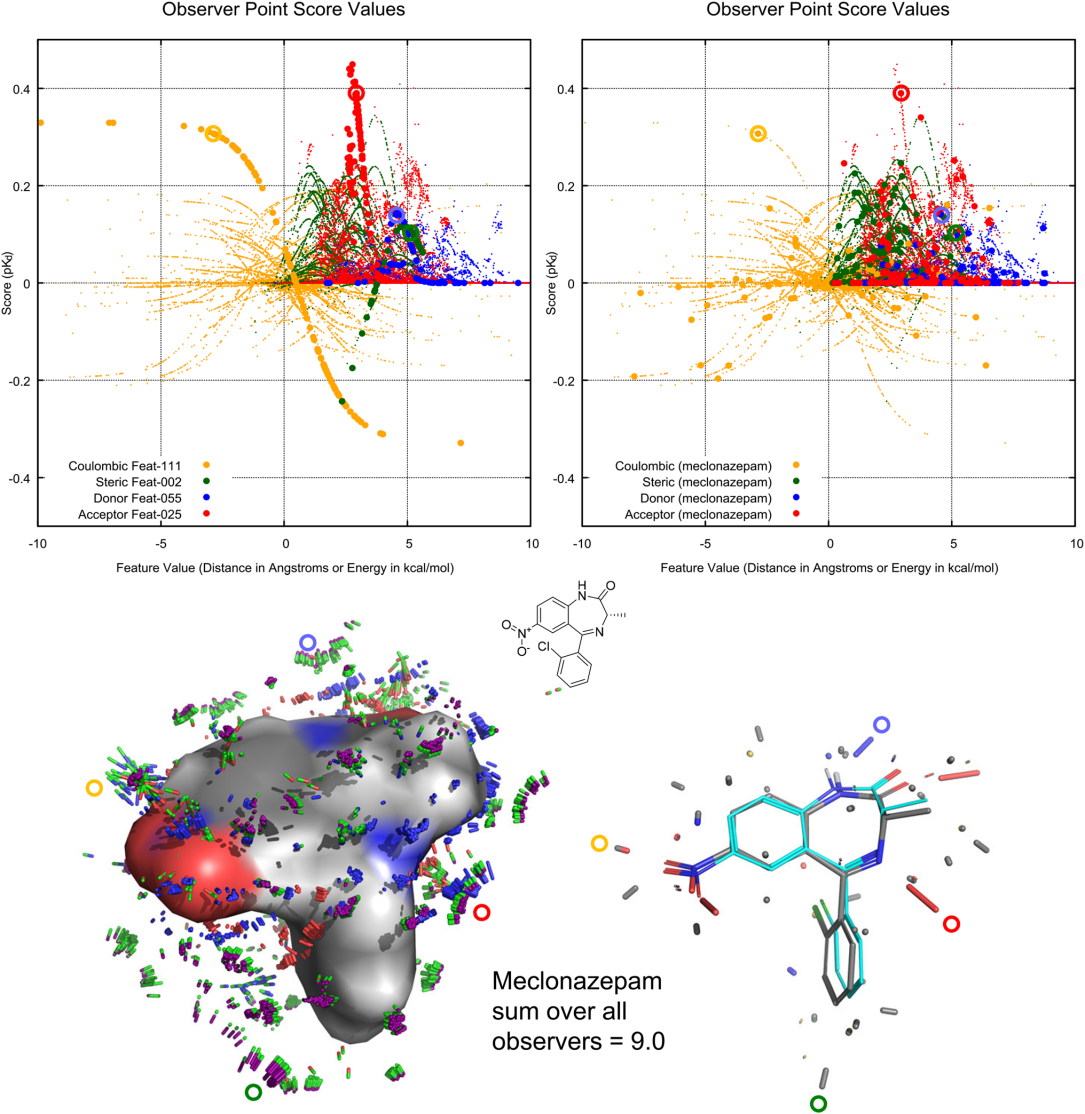
The learned response functions for each type of molecular feature at each observer point reflect the sigmoidal and Gaussian shapes of the underlying functional forms; bolded plot points (upper left) correspond to all observed values for one response function each for four observers over all training molecules; bolded plot points (upper right) correspond to all observer values for every response function for *only* meclonazepam; and the colored circles indicate the particular highlighted observers in the plots and the 3D depictions (bottom).

#### Overall QuanSA function

The overall predicted score *P* for a molecule in a particular ligand pose $L_p$ is given as follows:

10 $$\begin{aligned} P(L_p)&= \sum \limits _{i=1}^{n} (S_i + D_i + A_i + C_i) + strain(L_p) \end{aligned}$$

Recall that the individual response terms are each dependent on the particular ligand pose $L_p$, as is the strain value (described in detail below). The optimal pose for a ligand is that which maximizes *P*.

Figure 7 illustrates the learned response function values for the entire set of 98 BZR molecules in their final optimal poses at each of the 118 observer points (small dots colored orange for Coulombic response, green for steric, red for acceptor, and blue for donor). At top left, the response values are highlighted using larger dots for four different observer points, each having learned a key feature of activity. The large-amplitude orange sigmoid (one large dot for each training molecule) has learned a preference for negative charge, with its response inflection very close to zero charge (similar to the top left of Fig. 6).

Four particular points are circled, each corresponding to the particular value for the final optimal poses of training molecule meclonazepam. At right, large dots (118 for each of the four flavors of response function) represent the full set of values obtained by this pose. Each of the four circled values are close to the highest obtainable ones, and they correspond to the locations shown in the molecular projections shown below the plots.

At the bottom left is a depiction of the learned pocket field *z* and *p* values. Steric response function magnitudes are shown with half green and half purple sticks, with the length of the green portion reflecting $z^S_i$ and the length of the purple portion reflecting $p^S_i$. The red and blue sticks, respectively, represent receptor-side acceptors and donors, each seeking ligand-side complementation, with their lengths determined by $z^D_i$ (favorable to ligand donor) and $z^A_i$ (favorable to ligand acceptor). Sticks that are half-green and half red or blue (e.g. those near the orange circle) correspond to the magnitude of the Coulombic $z^C_i$ learned parameters. At the bottom right are atom-centric sticks, which integrate over the pocket-field’s interactions with the ligand. Note that final final optimal pose of meclonazepam (cyan carbons) is shifted from the initial pose (gray carbons) due to the optimization of molecular pose subject to the pocket-field and ligand strain.

The overall pocket-field function reflects a simple sum over geometrically interpretable local observations of a ligand taken from the fixed reference frame of the observer points plus a force-field based estimate of ligand strain (see Eq. 10). Because the response functions are non-linear, and because the score for a ligand is defined to be its maximum value over all possible poses, the pocket-field can represent complex phenomena such as size exclusion that lead to non-additivity of functional group contributions [21, 22].

### Observer point placement

The molecular alignment procedure (described next) produces potentially several alternative solutions, which consist of an ideal pose for each training molecule along with potentially many related alternative poses (see Fig. 2). As discussed above, the steric, donor, and acceptor observations are not overly sensitive to precise placement, but the Coulombic observations have a narrow range of distances in which they are properly responsive to changes in a molecule’s local electrostatic character. Because of this, the placement of observation points is made such Fig. that it is very likely that an observer exists within roughly 2–4Å of every part of the surface of any ligand that is in a reasonable pose. However, both to reduce the chance of overfitting and to reduce the computational expense, some care is taken to produce a *small* set of such points rather than simply producing everything within a rectangular grid having 2.0Å spacing.

An initial set of observation points is placed by first considering the optimal clique (one pose per training molecule) as the union of all constituent atoms. A sphere is placed with its center at the centroid of the clique, with its radius being 8.0Å beyond the furthest atomic surface point. This sphere is tessellated such that the inter-point distance close to 8.0Å. Each point is then moved as follows: 1) the minimum distance to the clique is calculated; 2) the point is moved toward (or away) from the centroid in order to make the minimum surface distance 2.0Å (so if the initial minimum distance is 10.0Å, the point will be moved 8.0Å toward the centroid); and 3) the process is repeated a total of ten times. This procedure very rapidly converges on a set of points, each of which is nearly exactly 2.0Å from the nearest atomic surface of the clique.

However, given a mixture of small and large molecules, it may be that some of the smaller ones are “hidden” by the larger ones and therefore do not have an observation point nearby. So, the process is repeated, but rather than using the merged set of all training ligands, each training ligand is processed individually. If any of the ligand-specific observer points is greater than 2.0Å away from the evolving set, it is added to the set.

The resulting observation point sets form the locations from which the feature values are computed that drive the QuanSA response functions. For the BZR case, the procedure results in 118 points, and for the steroid case, it results in 58.

### Initial ligand alignment

One of the most challenging aspects of fully automatic purely ligand-based 3D-QSAR is the problem of multiple ligand alignment. A key finding from our work on exploiting protein structures within QMOD to build better models was that the effect of greater fidelity in the initial ligand alignments had a much greater impact than specific details about the protein structures themselves [26]. While in a number of cases, *de novo* models have yielded solutions that are congruent with known binding sites [24, 25, 27], this is not always the case and, in general, depends on having multiple chemical series available during model-building. Here, we will briefly describe the more general QuanSA approach, which is built upon our prior work [24, 30, 37, 38]. A detailed study that includes results on larger benchmark sets for multiple ligand alignment is planned.

The QMOD implementation relied on a user to make a selection of two to four training ligands from which to develop an alignment hypothesis, against which all other training ligands were aligned. This choice could have a large effect on prediction quality, and the alignment procedure, by relying on a small number of molecules to guide the remainder, is limited in the degree to which it can produce an overall set of initial ligand alignments that will account for all of the molecular variation that may be present.

We have shown that one aspect of model-selection that is predictive of future performance is model *parsimony*. High-parsimony models are those for which pairs of training ligands that have similar activity levels also share high shape and electrostatic similarity in their optimal final poses, just as we see in the steroid example. We have incorporated the parsimony concept into the multiple alignment algorithm, making use of a clique score that strongly weights the similarity of pairs of molecules whose activities are similar: $w_{ij} = g((pK^i_d - pK^j_d),2)$. So, if a pair of molecules has activity values differing by 0.5 pK$_d$, the weight is 0.9, falling to 0.6 at a 1 log difference, and to 0.1 at 2 logs difference.

All-by-all mutual optimizations of flexible ligands become intractable quickly, but structure-activity data usually consists of many exemplars from discrete chemical series. So, mutual alignments are built incrementally from a core set of automatically chosen diverse exemplars. The procedure is as follows:

1. All N training molecules are subjected to thorough conformational search [28].
2. The M most different ligands are selected (using normalized 2D molecular similarity values [39]) from among the most active examples of the N training ligands. This is done as previously described with molecular diversity computations [40, 41]. The default value of M is 5, which is often enough to allow for thorough mutual similarity clique generation.
3. Given the M diverse ligands, each is flexibly aligned with each conformation of all others. The top scoring alignments are stored. Mutually similar core cliques of mutual alignments of M molecules are constructed by traversing through these pairwise alignments. All such cliques are scored based on pairwise similarity values weighted as described above, with the best such solutions being retained (and with redundancy removed based on RMSD). The default number of maximal of such cliques is 7, but fewer may be generated in the case of redundancy.
4. The best cliques of M ligands are completed by flexible alignment of the remaining (N-M) ligands (as well as generating additional alternatives for the M core ligands). Each of the molecules is aligned to each of the M conformers within the core clique, with the highest scoring poses being retained in a pose pool.
5. Each of the cliques now contains multiple poses for each of the full set of N training ligands. Using a greedy procedure, full near-optimal cliques of N poses are constructed incrementally. Given the final full N-molecule cliques, the pose pools are pruned such that no pose for any training ligand is excessively dissimilar from its optimal pose.
6. The final cliques along with their associated pose pools are sorted according to score, with each forming a possible alternative starting point for the QuanSA learning procedure.

This procedure automatically addresses the problem of ligand selection for core initial alignment construction as well as that of making use of the maximal context of data from many training ligands. Because full cliques are constructed greedily, newly added molecules are able to help guide the pose choices for later ones. Figure 2 shows the full 20-molecule clique for the steroid SHBG case along with the alternative poses for a particular molecule. Note that the alternative poses include ones with very high deviations in terms of RMSD from the optimal single pose, but that all poses are similar to one another in a 3D surface and electrostatic sense.

It is possible for a user to incorporate knowledge of the binding modes of some number of training ligands, by providing specific pose alternatives at the beginning of the procedure. It is also possible to specify constraints on the conformation of molecular subfragments or on the absolute position of a common element among different ligands. Such constraints are incorporated through the use of real-valued quadratic penalties imposed on deviations in dihedral angles or on specific atomic positions. In the work presented here, crystallographic docking-based guidance was used in two cases (AchE and thrombin), but no direct constraints on conformation or alignment were used.

For the steroid SHBG case (Fig. 2), the entire automated alignment process required less than 10 minutes.

### Parameter initialization and refinement

The preceding has described the response functions of QuanSA, the placement of observer points, and the generation of initial molecular alignments. Each observer point has nine associated learned parameters: $z^{S,A,D,C}_i$, $\mu ^{S,A,D,C}_i$, and $p^S_i$. QuanSA makes use of a parameter fitting regime that is biased toward finding a final optimal set of parameters such that the final optimal training ligand poses will be relatively close to the initial optimal clique.

**Table 1:** Summary of molecular datasets and their relative complexity.

Set name	Benchmark source	N train	N blind test	N ChEMBL test
Steroid globulins [1, 29, 32]	CoMFA/compass	21 (CBG), 21 (SHBG)	10, 61	–
5-HT1a receptor [23, 31]	Compass/QMOD	20	35	–
FEP benchmark [18]	FEP	199 (eight targets)	–	–
GABA$_A$ receptor [4, 27]	CMF/QMOD	98	49	1158
COX2 [4, 27]	CMF/QMOD	188	94	2308
AchE [4, 27]	CMF/QMOD	74	37	2436
Thrombin [4, 27]	CMF/QMOD	59	29	2947
Muscarinic receptor [21]	Pharmacia Med. Chem.	43 (refine: +26)	–	993

This is driven primarily by the initialization procedure for the learned parameters, which considers the single initially optimal pose for each training molecule:

1. The values for $\mu ^S_i$ are set to be the average steric distances to the ligands within the top window activity (default 3.0 log units).
2. The values for $z^S_i$ are increased uniformly from 0.0 until the minimum mean-squared-error between experimental and calculated activities (MSE) is achieved. The optimal value is stored, and $z^S_i$ are set to zero.
3. Initial values for $\mu ^D_i$ are set as with $\mu ^S_i$. Initial values for $\mu ^A_i$ and $\mu ^D_i$ are set in a similar manner, but the calculation also takes into account the directional magnitudes for the donors and acceptors (those with poor directional congruence to a particular observer point contribute little).
4. The values of $z^D_i$ and $z^A_i$ are adjusted in a similar manner as with $z^S_i$, simultaneously, again to minimize MSE.
5. The values of $z^S_i$, $z^D_i$, and $z^A_i$ are all set to half of their optimal values, producing an average model halfway between the steric-only one and the donor-acceptor-only one.
6. The values of all learned parameters (including the Coulombic ones) are then optimized using a gradient-directed line search, but the poses remain fixed to the initial optimal poses produced by the alignment procedure.
7. Finally, the values for all learned parameters are further optimized by gradient-directed line search. But now each pose for each molecule is considered, and the pose with maximal score defines the live pose for each training ligand.

This entire process takes very little time, just seconds for the steroid SHBG case. For this case, the mean absolute error (referred to as MAE or “mean error” in what follows) for the model with optimal uniform values of $z^S_i$ (Step 2) was 0.9 log units. For the model with donor-acceptor-only uniform $z^{D,A}_i$ values, mean error was 3.0 log units (reflecting the critical importance of hydrophobic shape in this case). The naively mixed 50/50 model (Step 5) produced an mean error of 1.7 log units. Line search with fixed poses (Step 6) produced mean error of 0.2 log units. Finally, in Step 7, allowing for model-based pose choice, the mean error dropped to 0.1 log units.

At this stage, a model is often quite accurate within the scope of the poses in the initial pose pool. However, when the model is applied, especially to molecules that diverge from the training set, one must optimize the poses of new molecules to the pocket-field that the model represents. Consequently, a final stage of model refinement is carried out, where iterations of model refinement are interleaved with full Cartesian pose optimization for all training ligands (subject to a strain calculation using the MMFF94sf forcefield).

In the steroid SHBG case, the mean error for the training ligands using the complete initial pose set was 0.1 log units. However, when the ligands are allowed to find optimal configurations, the mean error increases to 0.6 log units. In this simple case, a single iteration of parameter refinement produced a convergent solutions, with MSE of 0.03 and mean error of 0.2 log units. The entire parameter refinement procedure required just over 1 minute.

### Ligand strain

Ligand strain is important from two perspectives. First, clearly, highly strained ligand conformations should face some degree of penalty relative to less strained conformations when fit into a pocket-field in order to enhance predictive power as well as model specificity. Second, predictions of bound ligand poses that deviate strongly from what a trained computational or medicinal chemist knows to be reasonable conformations reduce confidence in a model.

From our recent work on the ForceGen method [28], we established that using the most thorough conformational sampling setting (-pquant, recommended for QuanSA), for non-macrocyclic ligands, a close-to-crystallographic solution ($\le 1.25$Å RMSD) is produced over 95% of the time. Initial conformer pools are restricted to 10 kcal/mol by default. Within the QuanSA scoring process, the global minimum that was uncovered by the thorough ForceGen search serves as a baseline. Deviations from this baseline energy (according to MMFF94sf) are weighted by a factor of 0.5. For example, a 1.0 kcal/mol deviation above baseline will result in the QuanSA score being reduced by 0.5 pK$_d$ units. This is just slightly lower than the normal conversion factor between units of pK$_d$ and kcal/mol.

### Prediction quality metrics

One critical aspect in contemplating predictions from any machine-learning method for binding affinity is that of the domain of applicability. A particular set of training molecules can only provide information on parts of a binding pocket where ligands have exhibited variation, both based on shape and electrostatic properties. QuanSA provides four semi-orthogonal measurements for each prediction: 1) novelty, 2) confidence, 3) envelope violation, and 4) raw nearest-neighbor similarity. The first three are probabilistically normalized values based on analysis of variation within the training set.

The normalization process makes it possible to compare different values between test molecules (and among different models). For example, for the confidence value, at the end of training, each molecule in its final optimal pose is compared to all other training molecules, and the maximally similar one is identified. The set of maximal similarities is used to estimate the parameters of a normal distribution, so that new molecules can be compared with the degree of variation seen in the training set. Where structural variability in a training set is low, a particular similarity score will be converted into a lower normalized confidence value than when the structural varaibility is high.

Novelty measures the degree to which a new molecule exhibits features that were covered by the full set of all training data (calculated based on distributions estimated from the training molecules). Envelope violation measures the extent to which a molecule protrudes from the envelope explored by the training set (see [27] for additional details on the envelop penetration computation). The raw similarity value is useful in cases where very limited structural variation exists in the training set.

### Molecular data sets

The results in this work were derived from the data summarized in Table 1. Each of these has either been a centrally important QSAR benchmark (e.g. the steroid and 5-HT1a sets), a challenging independently curated benchmark (the Sutherland Set, consisting of the GABA$_A$ (aka “BZR”), COX2, AchE, and thrombin cases), one that allows for direct comparison to a physics-based approach (the FEP Set), or a data set that offers particular insight into the application of ligand-based binding site modeling to medicinal chemistry lead optimization (the muscarinic set).

Together, the eight data sets cover a broad space in terms of target types, and they also cover a long history in 3D QSAR. With the exception of the muscarinic set, the data sets are described in some detail in the respective references in Table 1. The muscarinic set is analyzed in much more detail here than in prior work with QMOD [24]. The numbering scheme for the muscarinic antagonists used here was taken from the original reports, with series A numbering from Johansson et al. [42] and series B from Nordvall et al. [43].

### Computational procedures

The results reported here were generated using version 4.207 of the Tools and QuanSA modules of the Surflex Platform (the Docking module was also used in two cases). Ligand preparation was carried out as follows: 
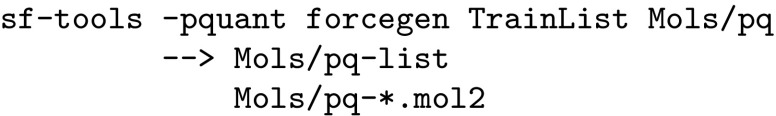
 This produced up to 1000 conformers for each training ligand (though typically far fewer), Details on the ForceGen methodology are presented in [28].

Initialization and model building was carried out as follows: 
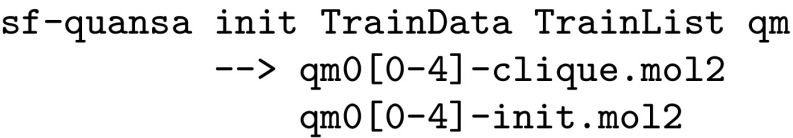
 The TrainData file contains activity information (e.g. mol-m4a = 10.0, and the TrainList file contains pathnames to conformer ensembles from the ligand preparation step (e.g. Mols/pq-mol-m4a.mol2. For small and relatively rigid ligand sets (steroid, serotonin, BZR, and COX2 cases), the option -clrms 0.5 was specified in order to produce additional initial alignment alternatives. For thrombin and AchE, poses derived from docking were used to guide the production of the initial ligand alignments: 
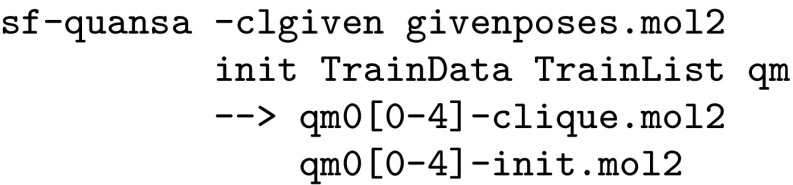
 Model building was carried out for the top five alignments produced by the initialization procedure. Two methods were used to aid in model selection, one which combined information from model induction (including parsimony quantification) and one where a cross-validation procedure was used: 
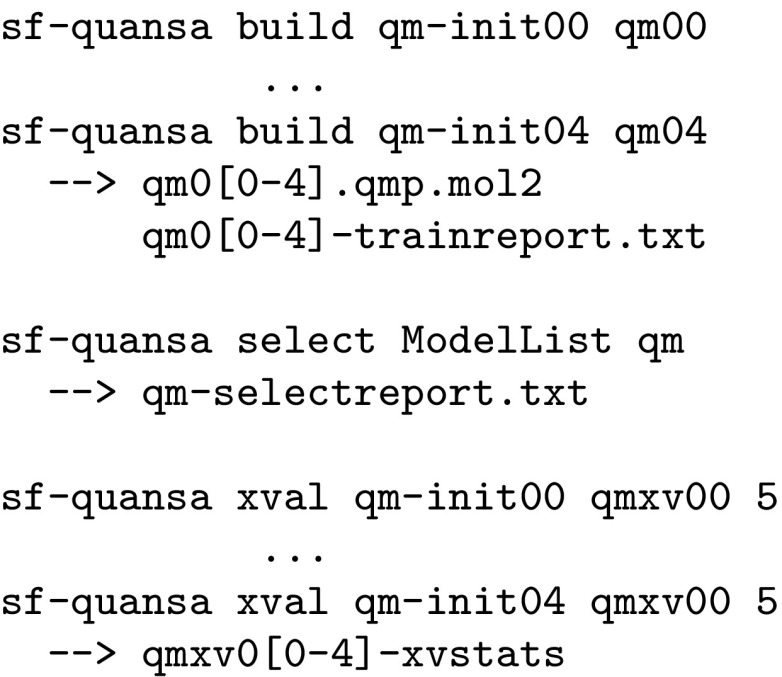
 Choosing a model based either on the best scoring using the select procedure or using that with the lowest MAE from the cross-validation procedure made little difference in blind tests within the benchmark sets (the two methods frequently agreed). Here, preference was given to the selection procedure that considered model parsimony.

For the muscarinic case, model refinement was carried out, using the following procedure for refinement and scoring: 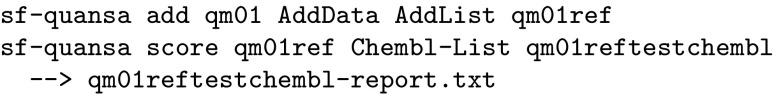 Additional details can be found in the data archive associated with this study (see www.jainlab.org for details on obtaining the data or the software).

## Results and discussion

Each of the data sets studied in the course of this work addressed different aspects of ligand-based binding affinity prediction. Results will be presented first on two sets of particular importance in the QSAR field [1, 31], then on multiple series that formed a validation benchmark for a recent implementation of a free-energy perturbation approach [18], then on four sets from a challenging QSAR benchmark [44]. Last, a series of muscarinic ligands is modeled, where the analysis is focused on the relevance of QuanSA to lead optimization [21] and on the concordance of the induced model to the recently solved crystal structure [45]. QuanSA models were generated purely from SAR data for SHBG, 5-HT1aR, the FEP data set, BZR, COX2, and the muscarinic receptor. To demonstrate the feature of using protein structure information during initial ligand alignment, the initial poses for AchE and thrombin were derived from docking.

**Fig. 8:**
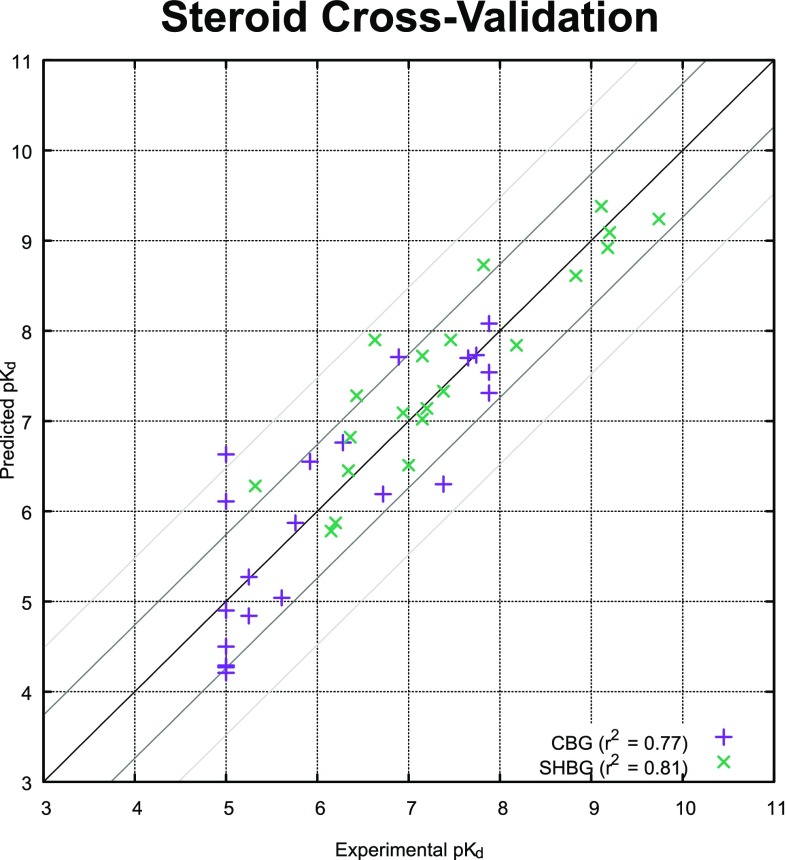
Leave-one-out cross-validation results for CBG and SHBG (lines indicate the 1 and 2 kcal/mol error boundaries).

### Steroid binding globulin and 5-HT1a${_R}$ examples

One very common approach to model quality assessment in QSAR has been cross-validation (often leave-one-out). With the QuanSA approach, cross-validation is offered as one of two means to adjudicate between alternative models, typically models derived from different initial alignments. The other model selection approach uses model’s quantitative parsimony along with the rank correlation and mean absolute error of the training ligands after having been re-fit to the pocket-field. For the CBG and SHBG cases, the latter approach identified the top-ranked alignments produced by the initialization procedure as being the preferred models. Figure 8 shows the results of a leave-one-out cross-validation for CBG and SHBG using the top-scoring initial alignment. In both cases, Pearson’s $r^2$ was very high (0.77 and 0.81, respectively). For context, the original CoMFA study, which introduced this benchmark, reported cross-validated $r^2$ values of 0.66 and 0.55, respectively [46], and a very early Compass study reported 0.89 and 0.88 [32]. It is important to understand that the 95% confidence intervals for the QuanSA $r^2$ results, owing to the small set of 21 molecules, were 0.44–0.92 (CBG) and 0.66–0.92 (SHBG). So, none of these results are likely to represent a substantial difference in predictive quality.

**Fig. 9:**
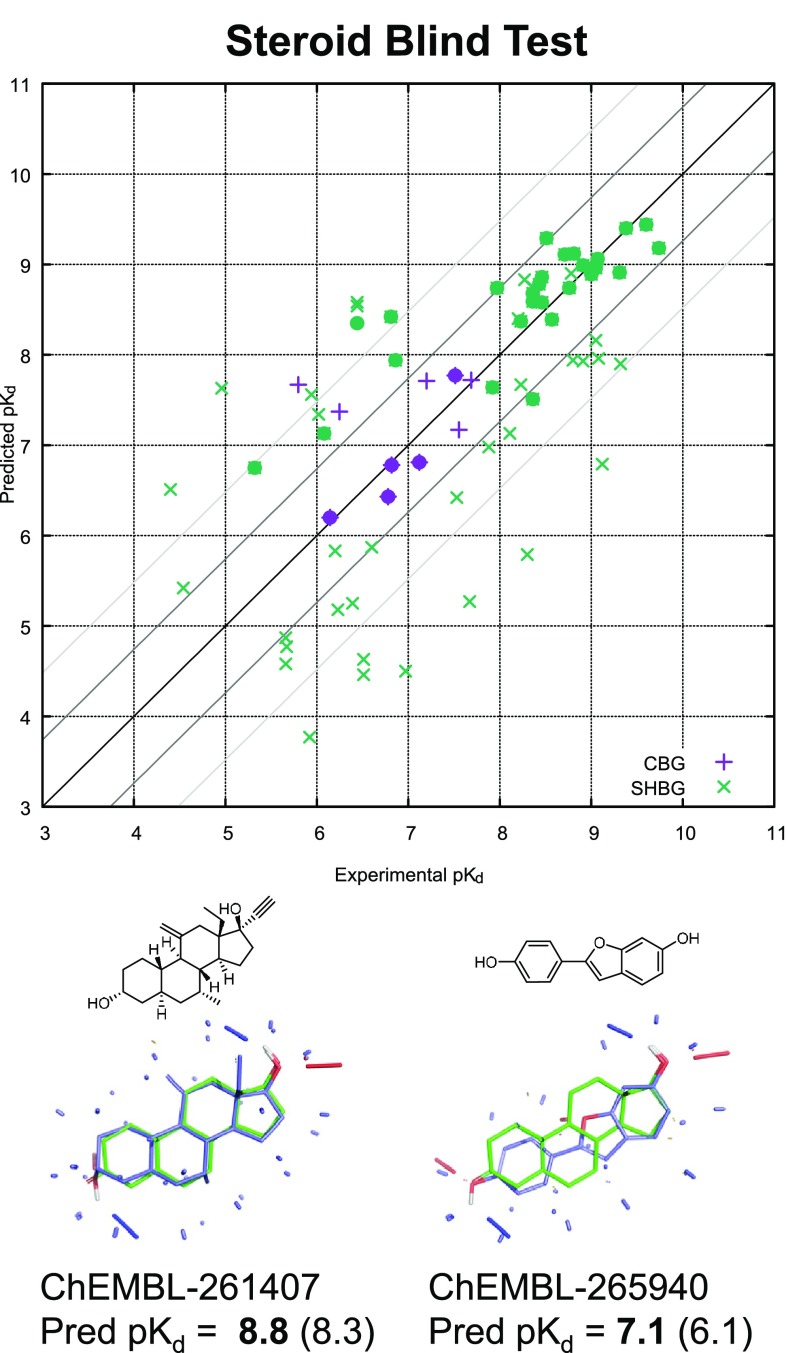
Blind prediction results for CBG and SHBG, with filled circles identifying in-model predictions.

Of course, cross-validation results are always less interesting than results on blind predictions (for any application of any machine-learning method). This is especially true for methods to be applied in lead optimization, where we expect to identify or design molecules that are different enough in character from training molecules to have different population characteristics [47]. Figure 9 depicts performance of the two QuanSA models on blind test sets consisting of 10 molecules for CBG (from the original CoMFA study) and 61 molecules from a much more recent study from Cherkasov et al. [29].

Based on prior work with QMOD, using the measures of prediction confidence described above, we adopted thresholds of $\le$ 0.85 for novelty, $\ge$ 0.35 for confidence, and $\le$ 0.95 for exclusion violations [27]. The conjunction of novelty and exclusion criteria form the broadest set of predictions that should generally be considered as likely to be accurate (termed “in-model”), and the conjunction of confidence and exclusion criteria usually produces a narrower set (termed “high-confidence”) with more accurate predictions. Note, however, that for predictions near the highest range of the training data (or higher), error magnitudes are typically quite low, independent of the quality measurements.

The highlighted points (filled circles) in the plot correspond to the set of high-confidence predictions. For CBG, the mean error for this set (just five molecules) was 0.2 log units, Kendall’s Tau (a non-parametric rank-correlation measure) was 1.0 (p < 0.05), and $r^2$ was 0.82. The ten-molecule CBG blind prediction set has been the subject of innumerable analysis, including particular discussion of an infamous fluoro-substituted outlier, where an H to F substitution produces a 2.0 log unit decrease in pK$_d$ (molecules 30 and 31 of the blind set). We believe that it should be incumbent on the prediction method itself to provide measures that allow for unbiased identification of the subset of molecule on which predictions should be believed. Further, prediction sets with just a handful of molecules are unhelpful in assessing model quality.

For the SHBG case, the blind test included 61 molecules, of which 27 were high-confidence (filled green circles in Fig. 9). For this set, the mean error was 0.5 log units, Kendall’s Tau was 0.74 (p < 0.00001), and $r^2$ was 0.67. The full set of 61 was sufficiently large and related enough to the training set (the mean raw similarity value was 0.90), that it is not unreasonable to consider prediction statistics overall: mean error 1.0, Tau 0.60 (p < 0.00001), and $r^2$ was 0.47. While quantitative performance clearly was diminished, the results were still highly statistically significant.

Figure 9 shows two blind predictions from the set of 61 diverse test ligands. The first (ChEMBL261407) met the confidence criterion but neither the exclusion nor novelty criteria. However, it was predicted to be among the highest activity values from the training set. The second (ChEMBL265940) met both the confidence and exclusion criteria despite being a substituted benzofuran rather than a steroid at all.

**Fig. 10:**
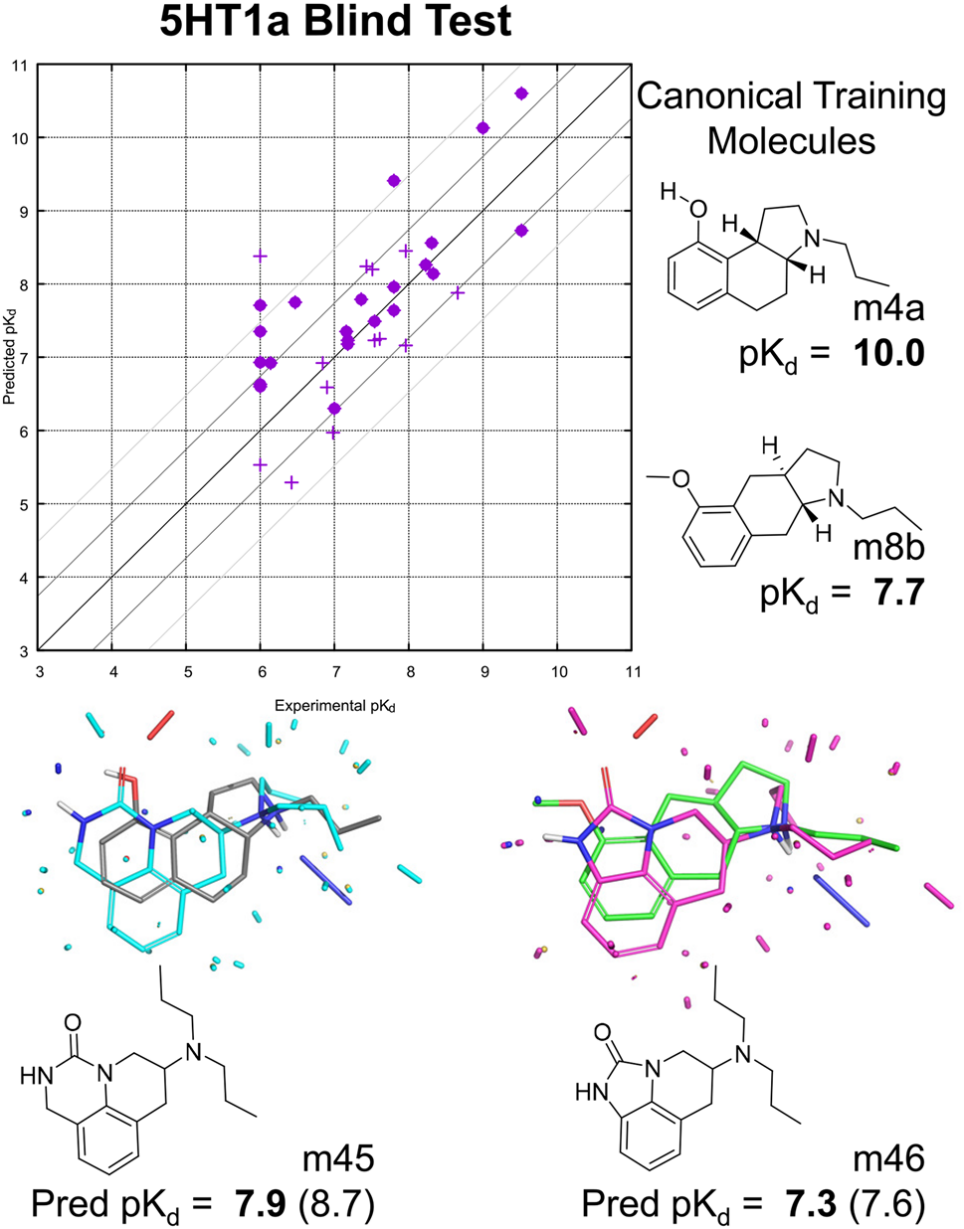
Training on 20 molecules, all with the canonical scaffolds shown above, produced a remarkably general model, as shown by a test of 35 molecules, including examples with very different scaffolds.

**Fig. 11:**
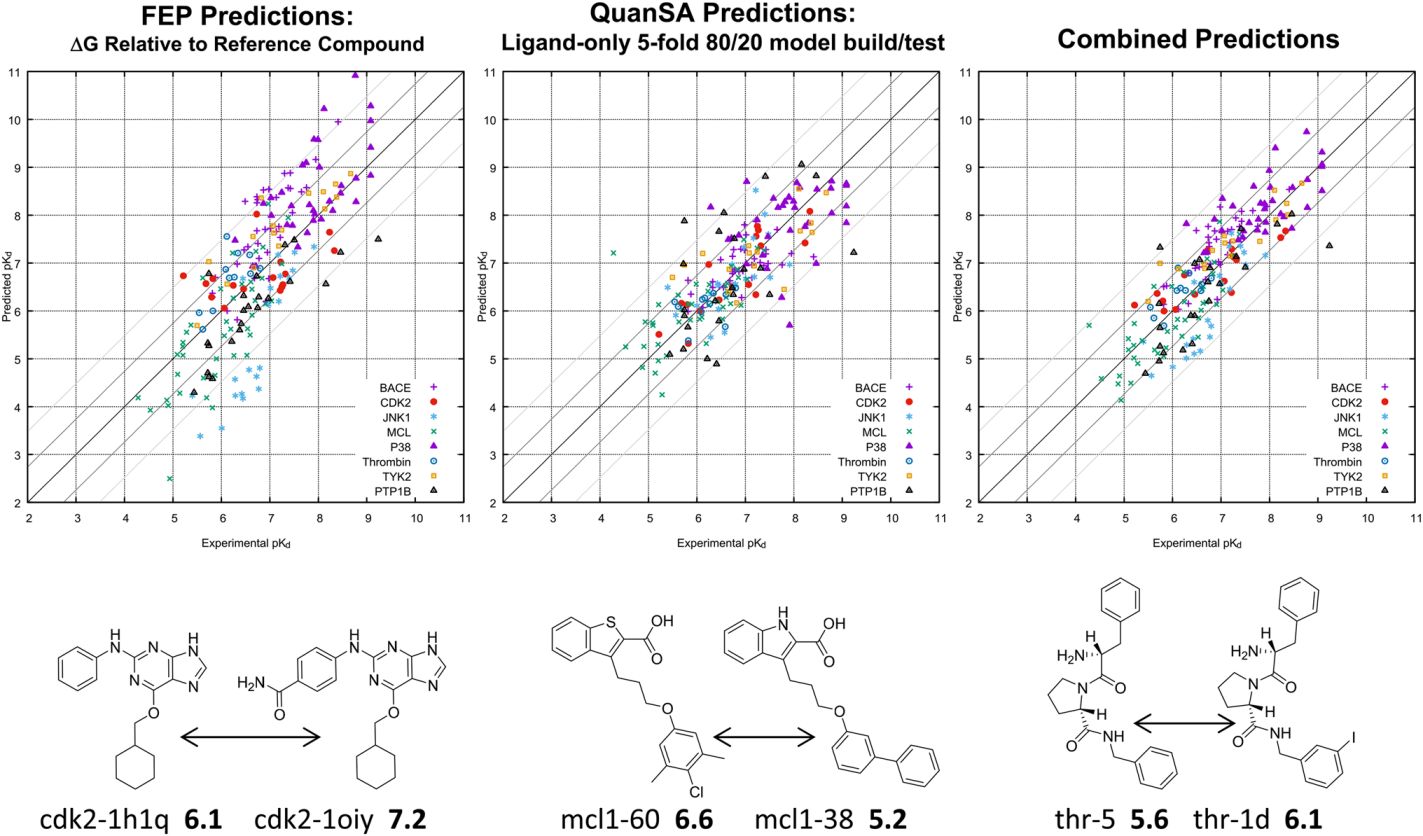
FEP calculated pK$_d$ using the specified target reference compound from which to calculate pK$_d$ for other target ligands using individual ${\Delta }{\Delta }G_{ij}$ calculations (left plot); QuanSA predicted pK$_d$ using purely ligand-based models (middle plot) constructed using 80% of the training data (repeated 5 times on non-overlapping splits), and the combined performance of the two approaches (right plot). Typical examples of FEP mutation pairs for three targets, with the left-hand compound in each case being the target’s reference ligand and the right-hand one having the largest change in experimental free energy of binding of those computed.

Figure 10 shows the results for constructing a QuanSA pocket-field using 5-HT1a ligands. This set of 20 training ligands and 35 blind testing ligands was originally used in a validation exercise for Compass [33]. The training set is exemplified by molecules m4a and m8b, and the test set consisted of variations in the angular and linear tricyclic structures (including changes to the ring fusion chirality) as well as more substantially different examples such as molecules m45 and m46. The benchmark is particularly challenging because the primary driver of potency variation is the detailed shape of hydrophobic parts of the ligands, with small changes in chirality or changes of a few atoms resulting in significant effects on pK$_d$. For example, the enantiomer of m4a has a pK$_d$ reduced by 1.5 log units, and the enantiomer of m8b has pK$_d$ < 6.0.

As seen in the plot, predictive performance was quite accurate, with most molecules (whether nominally in-model or not) being predicted within 1 kcal/mol and just a handful at or slightly beyond the 2 kcal/mol error level. For the 22 in-model molecules, Tau was 0.74 (95% CI 0.52–0.91, $p < 0.00001$), MAE was 0.64 log units. For the full set of 35 molecules, Tau was 0.58 (0.34–0.76, $p < 0.00001$) and MAE was 0.66. QMOD predictions for the full set of 35 resulted in a Tau of 0.34 ($p < 0.01$) and MAE of 0.8 [23]. Compass predictions produced a Tau of 0.36 ($p < 0.01$) and MAE of 0.8 [33]. The QuanSA predictions were clearly superior those from QMOD and appear to be significantly better than those from Compass.

The particular predictions shown in Fig. 10 on the novel scaffolds are not only quite accurate, but they appear to be correct for the right reasons. The pose optimization procedure placed the protonated amines in close correspondence to the training ligands, fit the alkyl nitrogen substituents into the pocket established by the training examples, and allowed the carbonyl of the urea to mimic the acceptor interaction made by hydroxyl substituents in various training molecules.

**Table 2:** Results on the FEP test set of 199 molecules under two prediction regimes for FEP and QuanSA (units for MAE are pK$_d$)

Target	N	FEP (corrected)		FEP (ref $\Delta {}G$)	QuanSA LOO		QuanSA 80/20 (fivefold)	
		Tau (95% CI)	MAE $\,\pm\, \sigma$	MAE $\,\pm\, \sigma$	Tau (95% CI)	MAE $\,\pm\, \sigma$	Tau (95% CI)	MAE $\,\pm\, \sigma$
BACE	36	0.66 (0.48–0.80)	$0.49\,\pm\, 0.40$	$0.90\,\pm\, 0.50$	0.51 (0.24–0.74)	$0.37\,\pm\, 0.33$	0.57 (0.37–0.74)	$0.35\,\pm\, 0.30$
CDK2	16	0.29 (− 0.16–0.71)	$0.65\,\pm\, 0.42$	$0.65\,\pm\, 0.43$	0.82 (0.53–1.00)	$0.38\,\pm\, 0.24$	0.78 (0.57–0.96)	$0.41\,\pm\, 0.24$
JNK1	21	0.87 (0.69–0.99)	$0.78\,\pm\, 0.33$	$1.30\,\pm\, 0.83$	0.68 (0.47–0.87)	$0.41\,\pm\, 0.31$	0.70 (0.52–0.86)	$0.48\,\pm\, 0.37$
MCL1	42	0.64 (0.49–0.77)	$0.62\,\pm\, 0.46$	$0.68\,\pm\, 0.53$	0.64 (0.43–0.81)	$0.39\,\pm\,0.41$	0.63 (0.39–0.81)	$0.44\,\pm\,0.48$
p38	34	0.53 (0.34–0.68)	$0.64 \,\pm\, 0.33$	$0.71 \,\pm\, 0.64$	0.41 (0.21–0.58)	$0.59\,\pm\, 0.37$	0.32 (0.07–0.55)	$0.66\,\pm\, 0.54$
PTP1b	23	0.78 (0.50–0.96)	$0.45\,\pm\, 0.40$	$0.74\,\pm\, 0.48$	0.59 (0.33–0.81)	$0.55\,\pm\, 0.58$	0.49 (0.19–0.74)	$0.82\,\pm\, 0.62$
Thrombin	11	0.60 (− 0.05 to – 1.00)	$0.31\,\pm\, 0.26$	$0.50\,\pm\, 0.42$	− 0.07 (− 0.89 to – 0.55)	$0.39\,\pm\, 0.35$	0.42 (− 0.25 to –0.74)	$0.32\,\pm\, 0.28$
Tyk2	16	0.80 (0.56–0.96)	$0.33\,\pm\, 0.27$	$0.50\,\pm\, 0.44$	0.72 (0.46–0.93)	$0.44\,\pm\, 0.28$	0.59 (0.28–0.87)	$0.56\,\pm\, 0.43$
All	199	0.68 (0.63–0.73)	$0.56\,\pm\, 0.40$	$0.77\,\pm\, 0.59$	0.72 (0.67–0.77)	$0.45\,\pm\, 0.38$	0.64 (0.57–0.71)	$0.51\,\pm\, 0.46$

Tau using the FEP reference molecule $\Delta {G}$ is the same as the corrected predictions in all cases, except for when considering all molecules, where Tau was 0.63 (95% CI 0.57–0.68))

The steroid and 5-HT1a benchmarks do not represent a comprehensive validation for any 3D-QSAR method. However, they represent a necessary condition. If a method cannot yield predictive models in these cases, where ligands are relatively small and rigid, and where molecular alignments are not enormously difficult, it is unlikely that more challenging cases of pharmaceutical relevance will prove to be tractable. Here, using fully automatic computational procedures, highly predictive models were produced with no requirement for manual ligand alignment.

### Physics versus machine-learning: comparison to FEP

As mentioned in the Introduction, there has been a resurgence in interest in practically applicable physics-based estimation of binding free energy, exemplified by a recent study [18], where data presented for 199 compounds covering eight pharmaceutically relevant targets enables a comparison here. The FEP approach typically requires a single ligand with known free-energy of binding along with a corresponding experimental structure of the ligand bound to the protein of interest. From this reference ligand, a set molecular transformations can be made and arranged into a connected graph such that connected pairs of molecules have relatively high similarity. For each such connected pair, a calculation of the ${\Delta }{\Delta }G_{ij}$ is carried out. To obtain a prediction for a particular molecule *k*, one begins from a molecule with known ${\Delta }G^{exp}$ and traverses a path of connected molecules, each time adding the calculated difference in energy. Cycle-closure constraints are enforced such that traversal of different paths to a particular molecule will yield the same value of ${\Delta }G^{pred}$.

Because of the construction of the connected graph of molecular mutations, one may initiate the calculation of any molecule’s ${\Delta }G^{pred}$ from any molecule whose experimental free-energy of binding is known. Figure 11 (left) shows the reported results for the FEP calculation in pK$_d$ units (top) for all eight targets. These results are for the case where the ${\Delta }G^{pred}$ values are computed from the single reference compound for each of the eight targets (a realistic application scenario).

The middle plot shows the results for QuanSA, where, for each target, five models were constructed, each with a non-overlapping 20% of the molecules reserved for blind scoring. No crystal structure information was used in any fashion. The pocket field models were induced using information only from the ligand structures and their corresponding activities. The collection of the five sets of blind predictions were used to assess performance. The FEP and QuanSA methods are essentially orthogonal in strategy, and their prediction errors are uncorrelated (data not shown). Consequently, the combination of the two, calculated by averaging each molecule’s two ${\Delta }G^{pred}$ values, results in an improved overall set of predictions (rightmost plot).

The three molecular pairs are typical of the molecular changes for which FEP ${\Delta }{\Delta }G$ values are computed and also give an idea of the variation within each target data set. The prediction conditions in Fig. 11 represent *realistic* scenarios for each method. For FEP, a connected set of alternatives are predicted from a compound whose bound structure and free energy is known. For QuanSA, information from a collection of compounds is used to predict on a set a quarter as large as that used for training.

Both methods also have *best-case* scenarios, each of which is worth analyzing. In the original paper, the reported FEP ${\Delta }G^{pred}$ were adjusted from what is shown in Fig. 11. Rather than calculating the nominal ${\Delta }G^{pred}$ values based only on the known reference ligand’s ${\Delta }G^{exp}$, the predicted free-energy values were re-centered such that their mean would match the mean ${\Delta }G^{exp}$ (the procedure is described in detail in the Supplemental Information for [18]). From a machine-learning perspective, this is spiritually similar to a leave-one-out validation experiment. For FEP, a cross-validation without any information contamination would have required making use of $(N-1)$ true ${\Delta }G^{exp}$ values to predict each “hold-out.” However, the mean estimated from $(N-1)$ ligands rather than *N* would not differ by much. The information leak from using ${\Delta }G^{exp}_{i}$ for a molecule in this fashion to calculate ${\Delta }G^{pred}_{i}$ is negligible. Note also that this correction has no effect at all on within-target correlation statistics.

For QuanSA, the best-case calculation is a leave-one-out cross-validation that makes use of the reference ligand’s bound structure to guide the induced molecular alignments. By leaving a single compound out, maximal information is used to derived the pocket-field, and ensuring that the ligand conformations are reasonable close to the correct absolute configuration provides a minor bias in favor of physically correct models (though these ligands and variations are simple enough that this makes little difference).

Table 2 shows summary statistics for rank correlation and MAE for both the best-case and realistic application scenarios for both methods. Here, Kendall’s Tau was used, with resampling-based calculation of confidence intervals (a value of 0.2 pK$_d$ units defined tied experimental activity values). For FEP, the prediction re-centering procedure improved the overall MAE by just over 25%, but in individual cases, the reduction in MAE was nearly 50%. For QuanSA, the MAE values are not substantially different, either per-target or overall. However, while the per-target correlation values have wide confidence intervals due to small sample sizes, the overall rank correlation performance begins to show an edge for the best-case approach. Due to small sample sizes, it is difficult to make a strong case, but it appears that the FEP approach is likely better in the thrombin case and that the QuanSA approach is better in the CDK2 case.

**Table 3:** Results from combining the FEP (uncorrected reference compound $\Delta {G}$) predictions with the QuanSA 80/20 pure ligand-based predictions

Target	Tau (95% CI)	MAE $\,\pm\, \sigma$
BACE	0.72 (0.54–0.86)	$0.46\,\pm\, 0.32$
CDK2	0.74 (0.46–0.97)	$0.41\,\pm\, 0.29$
JNK1	0.78 (0.62–0.88)	$0.73\,\pm\, 0.46$
MCL1	0.70 (0.55–0.83)	$0.44\,\pm\, 0.31$
p38	0.55 (0.33–0.72)	$0.49\,\pm\, 0.42$
PTP1b	0.71 (0.42–0.91)	$0.61\,\pm\, 0.44$
Thrombin	0.47 (− 0.16 to 0.89)	$0.30\,\pm\, 0.22$
Tyk2	0.85 (0.69–1.00)	$0.39\,\pm\, 0.31$
All	0.72 (0.67–0.77)	$0.49\,\pm\, 0.37$

The striking aspect of this comparison is not that one method is clearly better or worse than the other. Rather, it is that one method relies on direct simulation-based energetic modeling of protein-ligand interactions, that another infers the protein-ligand interaction energy landscape from structure-activity data, and that both methods produce very similar results across eight diverse targets. Further, the cancellation effect of orthogonal errors is substantial. Table 3 shows the rank correlation and MAE data derived by combining the realistic-scenario FEP and QuanSA predictions. In all eight cases, the combined result is either better than, or extremely close to, the best result from either method alone.

Using a single standard Intel i7 computing core, the QuanSA scoring process, in its default thorough search mode, required typically 20–40 seconds for molecules of the complexity represented by the FEP test set. By contrast, the FEP approach required approximately six hours per individual perturbation calculation using eight NVIDIA GTX-780 GPUs. As will be shown in what follows, QuanSA is suitable for large-scale calculations of thousands of synthetic alternatives, and it can be used to identify potent molecules with novel scaffolds. Where feasible, both computationally and in terms of theoretical applicability, methods such as FEP could be profitably employed to provide an orthogonal estimate of binding affinity.

### More challenging predictions and application to ChEMBL data

The FEP set includes eight diverse protein targets, but the ligand diversity is limited. As seen in Fig. 11, relatively small modifications at discrete positions on a single scaffold represented the typical range of structural variation. The 3D-QSAR benchmark reported by Sutherland et al. [44] was designed to present a significant extrapolative challenge for methods, with *designed* test sets for each of eight targets. For each target, approximately one-third of molecules for were selected by optimization using a maximum dissimilarity algorithm and were assigned to the test set, with the remaining compounds assigned to the training set. For four of the eight targets (BZR, COX2, AchE, and thrombin), substantial ChEMBL data was available, and this was used to further test extrapolative ability for the QMOD approach [27]. Here, we reports results for the QuanSA approach on these four targets using both the original blind test data as well as the ChEMBL data.

**Table 4:** Test results for the complete Sutherland benchmark

	QuanSA in-model				QuanSA full test			QMOD full test		
	%	$\tau$	MAE	$r^2$	$\tau$	MAE	$r^2$	$\tau$	MAE	$r^2$
BZR	45	0.54 (0.36–0.72)	0.54 (0.38–0.74)	0.39 (0.15–0.69)	0.53	0.61	0.36	0.42	0.65	0.27
COX2	81	0.54 (0.41–0.66)	0.84 (0.67–1.04)	0.41 (0.24–0.57)	0.49	0.90	0.34	0.39	1.01	0.22
AchE	68	0.58 (0.30–0.80)	0.71 (0.51–0.95)	0.57 (0.23–0.84)	0.51	0.83	0.47	0.60	0.68	0.56
THR	66	0.51 (0.19–0.77)	0.69 (0.50–0.89)	0.51 (0.18–0.77)	0.45	0.89	0.29	0.51	0.69	0.42

Table 4 summarizes results for these four targets on the Sutherland test set, with results for QuanSA included on the in-model subset of compounds as well as the full test set. In all four cases, QuanSA yielded statistically significant predictions for both the in-model subset and the full blind test ($p < 0.001$ for BZR, COX2, and AchE, and $p < 0.01$ for thrombin). The mean absolute error was in the range seen with FEP and QuanSA on the FEP Set under realistic prediction scenarios (with QuanSA error values being slightly lower and FEP error values being slightly higher). However, the Kendall’s Tau values were slightly lower for these four targets from the Sutherland Set. This primarily reflects the greater jumps from knowns to unknowns in the data underlying Table 4.

**Fig. 12:**
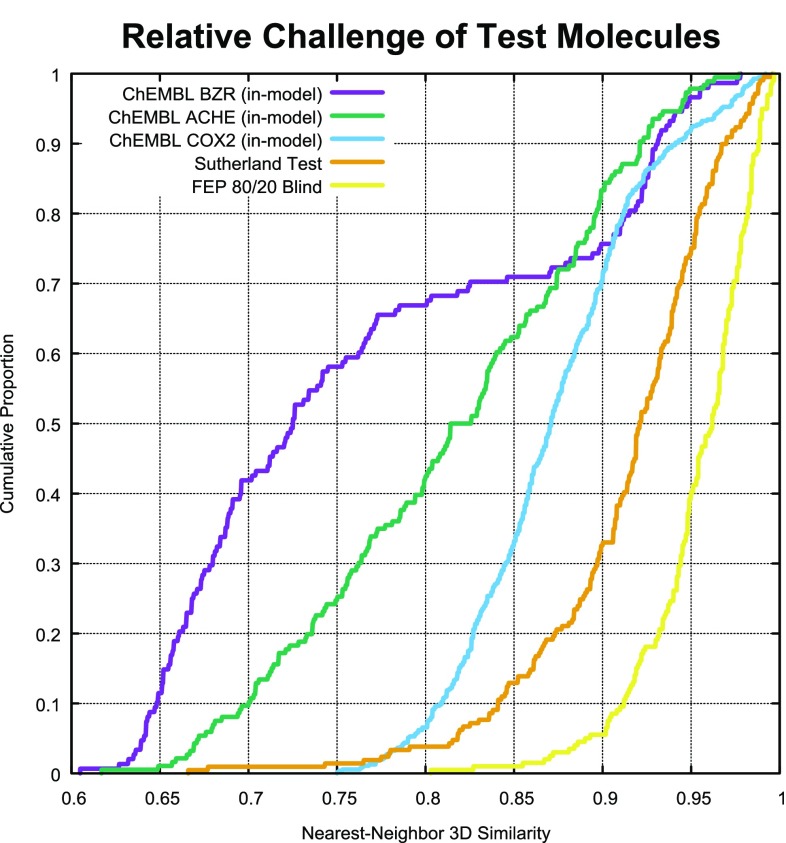
The relative difficulty of the molecules to be predicted varied considerably, as measured by the nearest-neighbor 3D similarity of the final predicted pose for each test molecule relative to the closest training molecule.

Ideally, it would be useful if one could compare Tau and MAE values from different methods on various data sets. The problem with such comparisons is that some datasets are exceptionally challenging relative to others. The relative challenge of each data set for prediction is quantified in Fig. 12. The median nearest-neighbor similarity for test molecules for the targets within the original Sutherland benchmark (orange line) was 0.92, which was substantially more challenging than the FEP Set (yellow line). For the 80/20 blind QuanSA validation for the FEP Set, 80% of the test molecules had nearest neighbor 3D similarities to training molecules of 0.93 or greater. For the Sutherland Set, fewer than 40% of the test molecules had nearest-neighbor similarities of that magnitude. Just 1% of test molecules within the FEP Set had 3D nearest-neighbors with 0.85 3D similarity or less, but the corresponding value for the Sutherland Set was 13%.

**Table 5:** Results for QuanSA models on diverse ChEMBL compounds, with N being the total number of tested compounds, “N i-m” being the number of in-model predictions, and the statistical performance assessed by Kendall’s Tau and mean absolute error

Target	N	N i-m	Tau (95% CI)	MAE
BZR	1158	148	0.25 (0.12–0.37)	1.2
COX2	2308	549	0.24 (0.18–0.30)	1.0
AchE	2436	186	0.26 (0.16–0.35)	1.6
Thrombin	2949	0	–	–
Muscarinic	993	291	0.34 (0.26–0.41)	1.1

Table 5 shows summary statistics for the four Sutherland targets on ChEMBL data (and for the muscarinic acetylcholine receptor, discussed in the next section). The total number of test compounds ranged from 1000–3000, and the coverage with respect to in-model predictions varied considerably. For the thrombin case, the training set was so small (59 compounds) and so narrow (all inhibitors were meta-substituted benzamidines with minor variations at two positions), that zero ChEMBL molecules met the criteria for being in-model. This case will not be discussed further.

For the other three cases, coverage ranged from roughly 10% for BZR and AchE to 25% for COX2, which had the largest training set (188 molecules, more than twice as many as the other cases). In all three cases, Tau was very similar (roughly 0.25), corresponding to very small p-values ($< 0.0001$). MAE was roughly 1 log unit for BZR and COX2 (less than 1.5 kcal/mol on average) and was higher for AchE (just over 2 kcal/mol).

Referring to Fig. 12, for ChEMBL predictions, the most similar in-model prediction set was for COX2 (blue line), with BZR being the most challenging (purple) and AchE falling in the middle (green). The AchE case presented additional difficulty due to the generally large and flexible ligands, which increase uncertainty with respect to pose and ligand energetics.

**Fig. 13:**
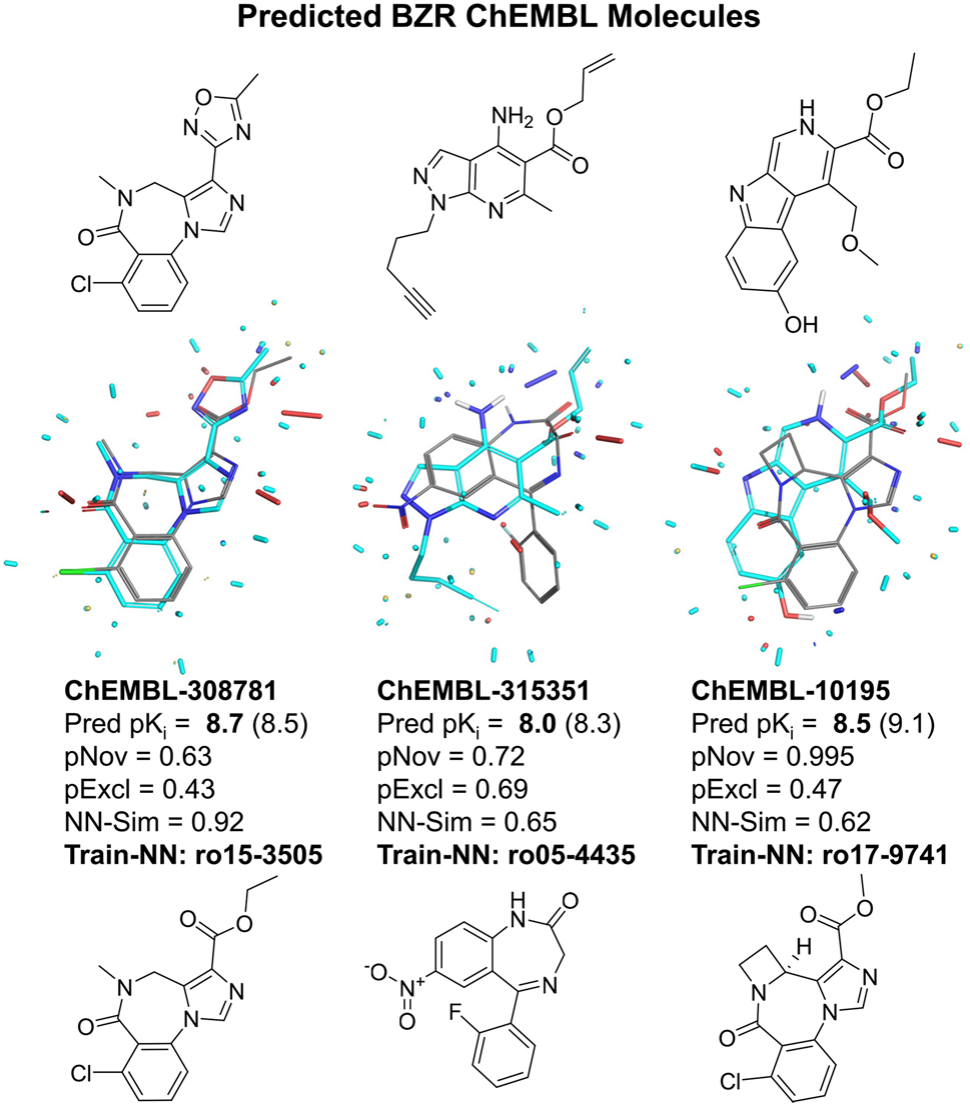
Examples of extrapolation to ChEMBL molecules for the QuanSA BZR model. The left-most and middle molecules are both in-model predictions, and the right-most falls above the novelty threshold.

Figure 13 illustrates two in-model ChEMBL predictions along with their nearest training neighbors along with one prediction whose novelty value exceeded the threshold of 0.85. The leftmost molecule was very accurately predicted, showing the introduction of a methyl-oxadiazole in place of the alkyl-ester that was well represented in the training data. From a medicinal chemistry point of view, this is an interesting leap, though likely not terribly surprising for those experienced with common bioisosteric substitutions [48]. For reference, the nearest-neighbor similarity in this case was 0.92, matching the typical test molecule from within the Sutherland Set.

The canonical BZR ligands used to construct the QuanSA model were known from the early 1960’s (e.g. diazepam [49]), the early 1970’s (e.g. alprazolam [50]), and the early 1980’s (e.g. compounds such as RO 15-3505 [51]). The middle molecule, representative of several well-predicted pyrazolo-pyridine esters, is a genuinely significant scaffold leap. The scaffold was first disclosed in 1989 [52] and was designed on the basis of a similar triazolopyridazine known from about a decade earlier. The rightmost molecule, a carboline derivative that is out-of-model, is representative of a class that was known contemporaneously with some of the training molecules, but none were included in the training set.

These QuanSA predictions are significant for four reasons. First, the GABA$_A$ receptor is a complex hetero-multimeric ligand-gated ion channel, part of a large group of important pharmaceutical targets that are very challenging for biophysical characterization down to atomic resolution. The pocket-field was constructed automatically, using only ligand structure and activity information. Second, its prediction quality rivaled that seen for the FEP Set, where protein structures were known and where structural jumps were smaller. Third, because the method is relatively fast, application to thousands of candidates (using modest computer hardware) is possible, allowing for evaluation of a large chemical design space, with built-in calculations for the applicable model prediction domain. Fourth, the method produced predictions of both pK$_d$ *and* bound pose, with the latter offering support for predictions that otherwise might be difficult to justify for experimental follow-up.

**Fig. 14:**
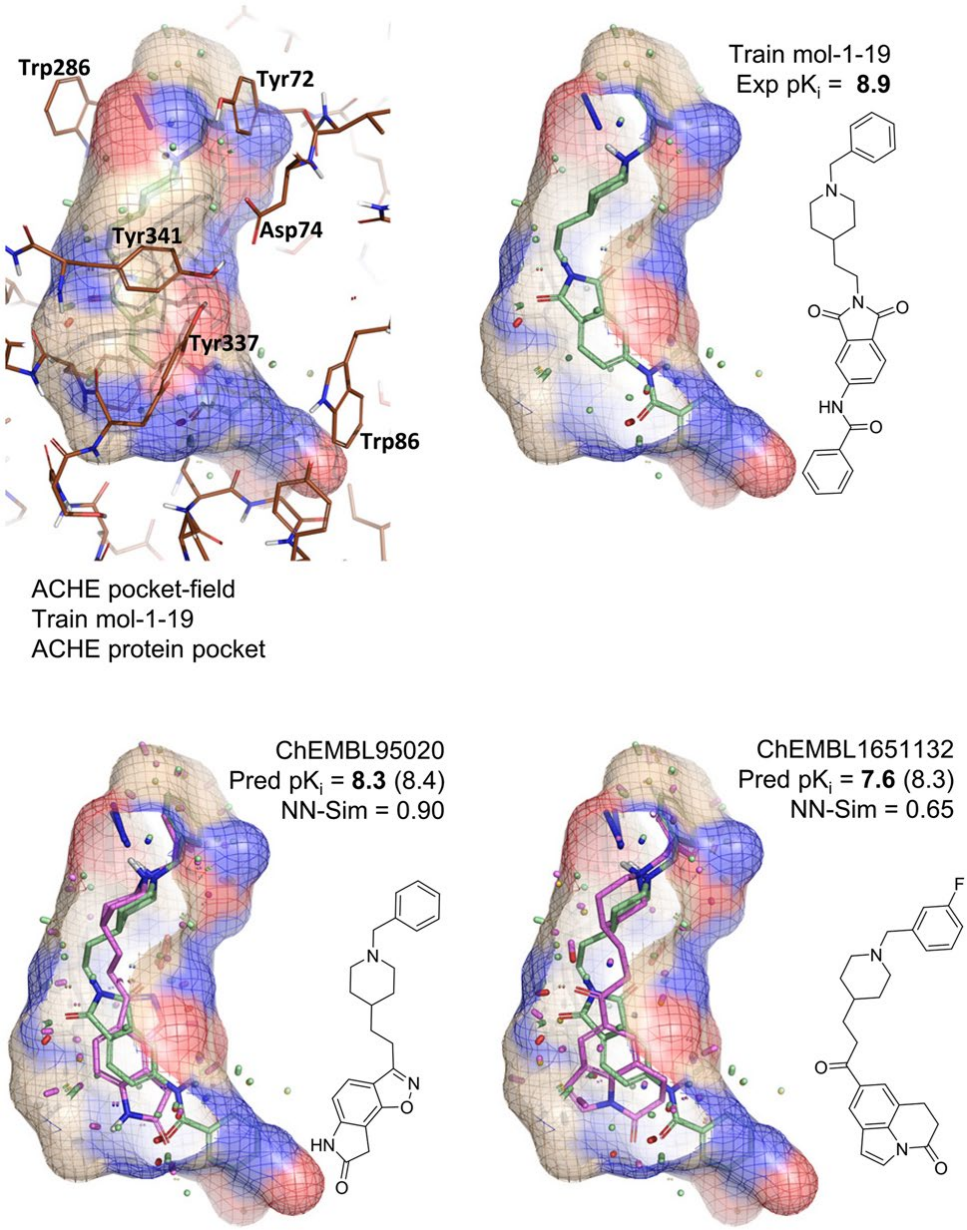
Examples of extrapolation to ChEMBL molecules for the QuanSA AchE model.

**Table 6:** Test results for the complete Sutherland benchmark.

Target	Full ChEMBL set			QuanSA in-model pred. $pK_i \ge 7.5$						1000 Decoys
	N	$pK_i \ge 7.5$	$pK_i \le 6.5$	N	Mean $pK_i$	N TP	TP %	N FP	FP %	FP %
BZR	1158	309	544	90	6.9	38	12.3	32	5.9	1.1
COX2	2308	351	1488	169	7.2	90	25.6	49	3.3	0.0
AchE	2436	491	1437	36	7.6	24	4.9	14	1.0	0.0
Musc.	993	350	427	66	7.2	31	8.9	17	4.0	0.0

Figure 14 illustrates the AchE QuanSA pocket-field. The AchE enzyme pocket is a 20Å gorge, with key interactions being made by the labeled residues. In particular, Trp286, Tyr72, Asp74, and Tyr341 of the peripheral anionic site play a key role in the electrostatic attraction to cationic ligands. Tyr337 and Trp86 near the bottom of the gorge interact with the quaternary ammonium group of the substrate acetylcholine. The transparent surface shows the induced pocket-field, and the amine of training molecule 1-19 can be seen to have a strong interaction in the direction of the face of Trp-286 (long blue stick). Lower down, the dual carbonyl atoms of the phthalimide linker make favorable interactions (red sticks) with both sides of the pocket (blue shaded area).

ChEMBL-95020 presents a convincing superimposition of the isoxazole-containing tricycle [53] to the phthalimide of the training molecule. In a 2D sense, this prediction seems more surprising than the nominal 3D similarity value would suggest, but the actual disposition of chemical functionality and its mimicry of known ligands makes this not only an in-model prediction but a *confident* one that is also accurate. The right-hand prediction [54] in Fig. 14 is also in-model, but it is of lower confidence and is more typical of the level of prediction error in the AchE ChEMBL set. Rather than making very similar polar interactions as the phthalimide, it fills the lower-left hydrophobic cavity of the pocket. However, the areas of correspondence between the novel ligand and in terms of pocket-field complementarity and relationship to other AchE ligands provides confidence in the prediction.

Many more examples of such extrapolative predictions exist, but rather than enumerate them, Table 6 provides summary statistics on true positive recovery rates and two different estimates of false positive rates. Here, we have defined true positives as those molecules for which pK$_i \ge 7.5$. For those molecules with measured activity, false positives were defined as those molecules for which pK$_i \le 6.5$. This is a strict test of the ability to distinguish relatively active molecules from those that are slightly less active, and it is relevant in contemplating the effectiveness of a prediction method for synthetic prioritization. We have also made use of a decoy set of 1000 drug/lead-like ZINC molecules, with the entire set presumptively defined as false positives. Considering the FP rate for the decoy set the value of a predictive method when evaluating very large numbers of candidates, possibly from a virtual screening library or a computational *de novo* design procedure.

For COX2, where we have not shown specific examples, the coverage of the ChEMBL molecules was highest (about 25%), and the relative similarity of the in-model predictions to the training set was also high (see Fig. 12). This lead to a true-positive rate from the full ChEMBL set of 26%, so a model constructed from fewer than 200 COX2 inhibitors (utilizing no protein structure) was able to identify a quarter of all of the potent COX2 inhibitors curated within ChEMBL, representing many years of discovery efforts. The enrichment rate was 8-fold (known actives to known inactives). From the 1000 ZINC decoys, not a single molecule was identified as an in-model predicted true positive, suggesting an enrichment rate in a screening scenario of well over 2000-fold. The situation for AchE was very similar, albeit with a lower true-positive rate 5%. Again, however, the model was very specific, with a 5-fold TP/FP enrichment between known actives and known inactives and not a single false-positive decoy identified.

The BZR case showed true-positive identification between that of AchE and COX2 (12%), but the false positive rate on the known inactive molecules was higher, leading to an enrichment of 2-fold. Similarly, application of the pocket-field to the decoy set identified 11 decoys, suggesting a screening-oriented false-positive rate of 1% and an enrichment of just over 10-fold. It is not clear whether the BZR model is truly less accurate or less specific than the others, though this seems somewhat unlikely based on assessment of its quantitative predictive accuracy. It may be the case that the decoy set inadvertently contains a number of *bona fide* BZR ligands. The nominal nearest-neighbor similarities of the 11 nominal false-positives ranged from 0.67–0.90, which is within the range observed for the in-model ChEMBL predictions, but this is not definitive. It may also be the case that the BZR binding site is truly significantly more promiscuous than the other binding sites, but we have no direct evidence of this.

### Summary of comparative performance

Two sets of comparisons to other methods have been carried out. The first set was to other QSAR methods on benchmarks of wide use or of particular significance to related methods. The second set was to FEP, a sophisticated physics-based simulation approach.

The QSAR comparisons included two steroid binding globulins [1, 32], 5-HT1a receptor, and four targets from a more recent benchmark [4, 44]. Considering the limitations of assay quality and of data set size, QuanSA performed as well or better than any reported method (including CoMFA and related methods and Compass) on the classic steroid cases, including accurate extrapolation to structurally novel molecules published 20 years after the introduction of the sets [29]. The 5-HT1a receptor case was the central validation example for the Compass method and was also used for validation of the initial QMOD approach [23, 33], and QuanSA performance was superior to either method. In the most challenging of the Sutherland cases (BZR and COX2), QuanSA results were substantially better than those seen with QMOD, whose performance exceeded that of CMF, CoMFA, and numerous 2D methods [4, 44].

With respect to the FEP comparison, individual chemical mutations across the entire set of eight targets resulted in errors of 0.9 kcal/mol for FEP [18]. For QuanSA, the comparable situation was where 80% of the data was used to induce a purely ligand-based model to then predict the remaining 20% of the data, and the result was a mean error that was slightly lower (0.7 kcal/mol). More importantly, though, was the observation that the errors made by the two methods were uncorrelated, so that combined predictions achieved robust predictive performance across all eight targets.

### Explanatory power and correspondence with future crystallography

A particularly interesting aspect of drug-design for older targets that lacked biophysical characterization for many years is that chemical exploration was done agnostically, with the synthesized chemicals themselves being used to elucidate binding pockets. Consequently, exploration of positions on a particular scaffold were often driven by considerations of systematicity and synthetic feasibility.

**Fig. 15:**
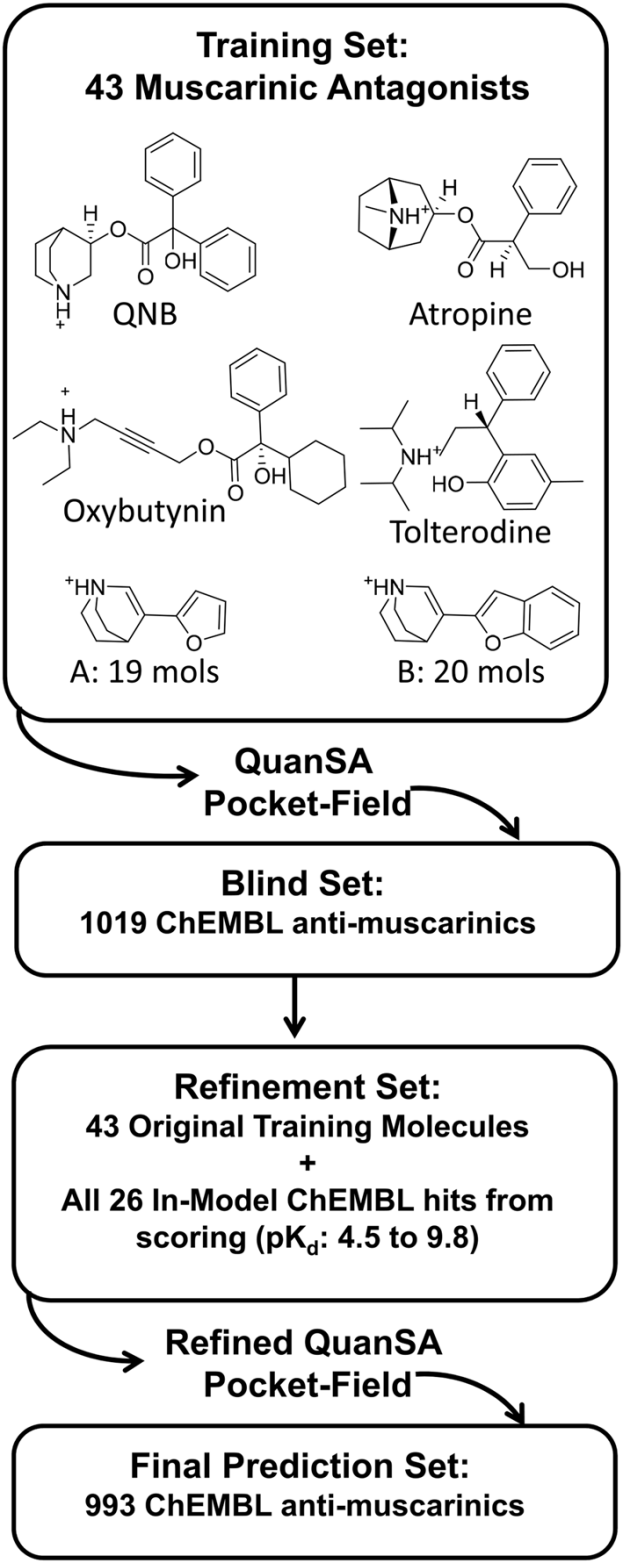
Muscarinic model training, refinement, and scoring procedure.

The Compass method, an antecedent to QuanSA, was developed at Arris Pharmaceutical and was refined during a period of collaboration between Arris and Pharmacia in the early to middle 1990’s. This was a period during which Pharmacia was also pursuing muscarinic antagonists, resulting ultimately in the approval of tolterodine by the US FDA in 1998 (see Fig. 15). At the time, the potent anti-muscarinic QNB was commonly used as a radioligand for displacement assays, and oxybutynin was a competing muscarinic antagonist. Of course, atropine as a medicinal compound had been known for many decades, and it was established as a potent muscarinic antagonist in modern pharmacological assays by the 1950’s [55]. During this time-period two series of quinuclidinene anti-muscarinics were pursued by Pharmacia [42, 43].

Figure 15 depicts a QuanSA model-building and refinement process that made use of a total of 43 training molecules (the four named molecules above plus 39 from two quinuclidinene series, with various substitutions on the furan and benzofuran heterocycles and including variations such as thiophene and benzothiophene analogs). The process was completely automatic, using default parameters for ligand preparation, initial alignment generation, and model building. The model selection procedure identified the second-ranked alignment clique of the top five as being likely to be most predictive. The resulting pocket-field was used to score 1019 ChEMBL molecules, of which just 26 were nominally in-model. Given the poor coverage, these 26 molecules (actual pK$_i$ ranging from 5.3–10.5) were used to refine the model.

**Fig. 16:**
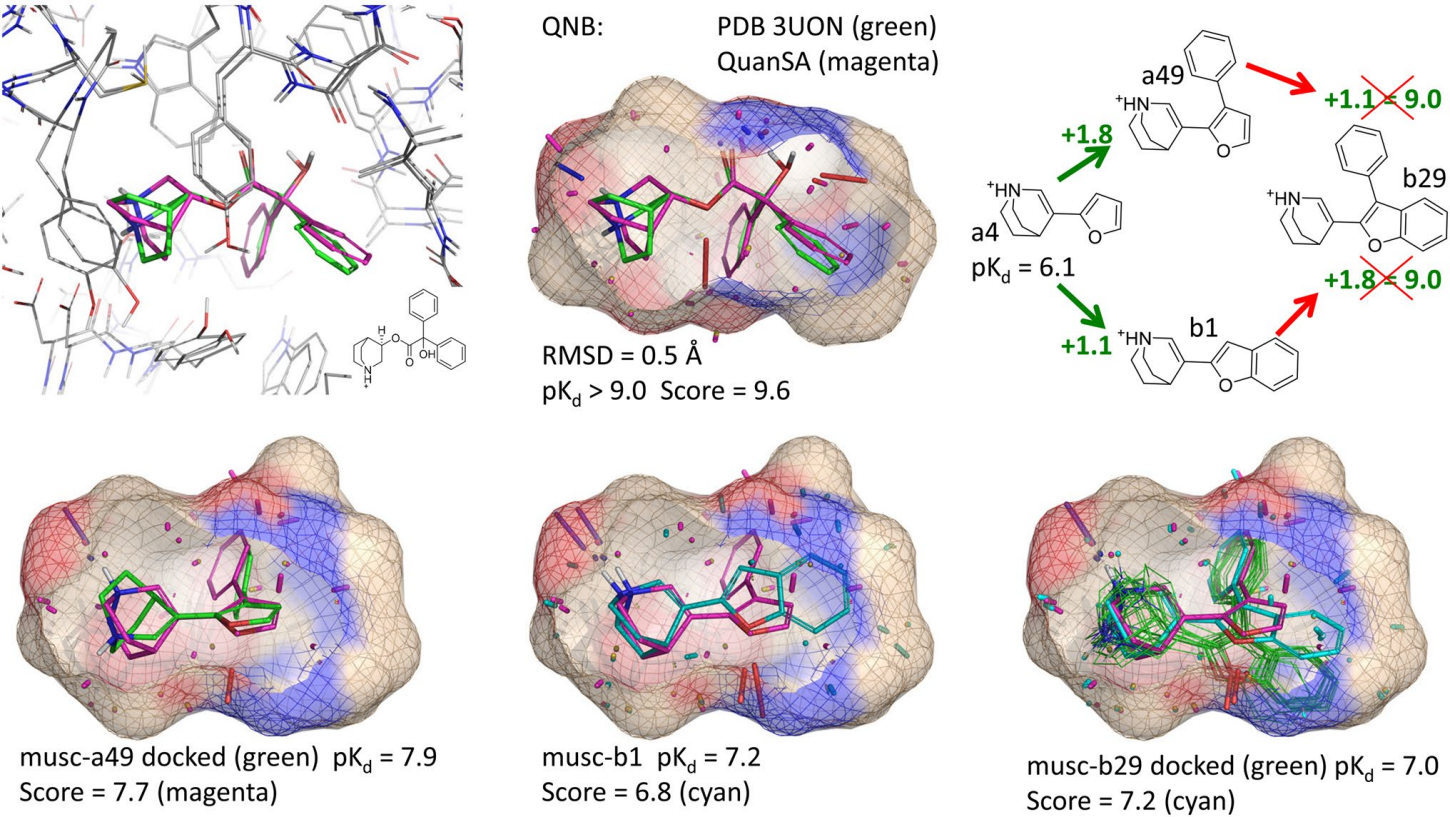
The human M2 receptor bound to QNB aligned with rat M2 and the QuanSA predicted conformation of QNB (top left); the pocket-field and interactions with QNB (top middle); a striking example of substituent effect non-additivity (top right); and the predicted poses by docking (a49 and b29, green) and QuanSA (a49 in magenta, and b1/b29 in cyan).

Apart from statistics of quantitative accuracy, the value of a physical QSAR model derives in part from the degree to which it is making predictions for the right reasons. As with the steroid globulin case presented first, the decades have produced critical crystallographic information. In particular, PDB structure 3UON revealed the bound configuration of QNB bound to the human muscarinic acetylcholine receptor [45], which is the biological target that was being investigated heavily roughly 20 years earlier. Figure 16 shows the protein binding pocket bound to QNB along with another antagonist-bound variant (rat M3, PDB Code 4U15). The crystallographic data was aligned to the muscarinic pocket-field using the ligand QNB to minimize automorph-corrected RMSD.

The predicted conformation of QNB (magenta, top middle of Fig. 16) was just 0.5Å RMSD from the bound form (green). The orientation of the amine was slightly off, and the orientation of the hydroxyl was significantly rotated away from the clear preference of the bound form. When bound, the hydroxyl proton interacts with the carbonyl of Asn-386. The predicted orientation is driven by energetic preferences of the ligand, turned toward the QNB’s ester carbonyl. This is similar to the problem of hydroxyl rotamers seen in the steroid globulin case (Fig. 4). Very often, within a collection of SAR data, there will be no information that allows for an unambiguous determination of the absolute conformations of the molecules. In such cases, this will become a problem when a molecule to be predicted *would* resolve the ambiguity but where the model has guessed incorrectly. Nonetheless, the 0.5Å deviation represents excellent agreement, given that QNB is a reasonably flexible molecule.

The two quinuclidinene series shown in Fig. 16 were being optimized for potency, with the benzofuran scaffold yielding no significant improvement over molecule b1 (pK$_d = 7.2$), despite extensive synthetic effort [42]. However, exploration of the furan scaffold yielded significant improvement (e.g. a49, with pK$_d = 7.9$, essentially equipotent to tolterodine). Variations such as b29 (the analogous phenyl variant of the benzofuran scaffold to a49) represent particularly challenging data points to explain. Considering the 2D SAR, and assuming additivity, b29 should have been a significant improvement over b1 and a49, but it was essentially equipotent with b1.

To understand the reasons for the puzzling SAR, and to see whether the QuanSA approach would help explain it, we compared the results of docking (green sticks) to predicted pocket-field poses (magenta for a49 and cyan for b1 and b29). For a49 (lower left), there was good agreement between the QuanSA-predicted binding mode and that from docking, though due to the rigidity of the molecule, the orientation of the quinuclidinene was non-optimal in the docking. The 3-phenyl substituent fills the back of the pocket, and the furan oxygen points forward, making a favorable pocket-field interaction. In the actual binding pocket, it appears to be a mobile tyrosine hydroxyl (clipped from the front of the protein pocket depiction) that interacts with the furan oxygen.

Molecule b1, the unsubstituted benzofuran (cyan), orients its oxygen downward in order to optimize its pocket-field interaction, in direct conflict with the required orientation for a49 (magenta). Docking of b1 (not shown) resulted in many possible fits, all of which were inconsistent with the docked pose of a49, but some of which were close to the preferred QuanSA pose. For molecule b29, the anomalously less active phenyl benzofuran, the QuanSA-predicted pose (magenta, lower right), shifted the aromatic ring down from the preferred position of a49. The docking of b29 shifted the ring downward further, but in qualitative agreement with the model’s prediction. Here, there are clearly conflicting preferences for the phenyl substituent, the furan/benzofuran oxygen, and the hydrophobic portion of the benzofuran. The QuanSA model was able to induce a model of the binding pocket that quantitatively explained the activities and qualitatively explained the reason for the non-additive behavior.

Non-additive SAR is actually quite common, as pointed out by Klebe’s group [22]. Using detailed thermodynamic and crystallographic data involving thrombin inhibitors, they explicitly showed that a particular functional group change (-H to -NH$_2$) exhibited context dependent ${\Delta }{\Delta }G$ effects. In cases where addition of the amino group created a “conflict-of-interest” with respect to the preferred binding mode of the sister molecule, the relative improvement of binding affinity was decreased. Strict additivity of functional group contributions to binding should be expected only when the functional group modifications *do not* affect the conformation of the rest of the molecule or its overall alignment (and even in those cases may be affected by more subtle effects on free-energy such as differential enthalpy/entropy compensation).

The initial, unrefined, pocket-field had a mean absolute error of 1.4 log units on the 26 in-model molecules, and the Kendall’s Tau of 0.19 missed a reasonable cutoff of statistical significance ($p = 0.12$). However, the refined model (constructed from a total of 69 muscarinic ligands) covered 291 (29%) of the 993 remaining ChEMBL muscarinic set. The Kendall’s Tau rank correlation was 0.34 ($p < 10^{-6}$) and MAE was 1.1 log units (see Table 5). The model was also extremely specific in terms of decoy rejection (see Table 6), and it was comparable in quality for rejection of known inactives to the COX2 model.

The overall true-positive identification of was 9%. This appears modest (a random selection of 66/993 would produce approximately double that number), but in the context of the false positive rates, the predictive value is clear. The QuanSA-driven procedure identified twice as many actives as inactives from the ChEMBL set, despite the *a priori* probability being in the other direction. For the ZINC decoys, the random selection process would have yielded roughly 70 false positives, but the QuanSA calculation produced zero. It would require much larger decoy sets to establish an accurate false positive rate in a screening sense, but based on the distributions of scores, novelty values, and exclusion values, it is likely to be less than 1/10,000, which suggest an enrichment rate of 1,000-fold or greater.

**Fig. 17:**
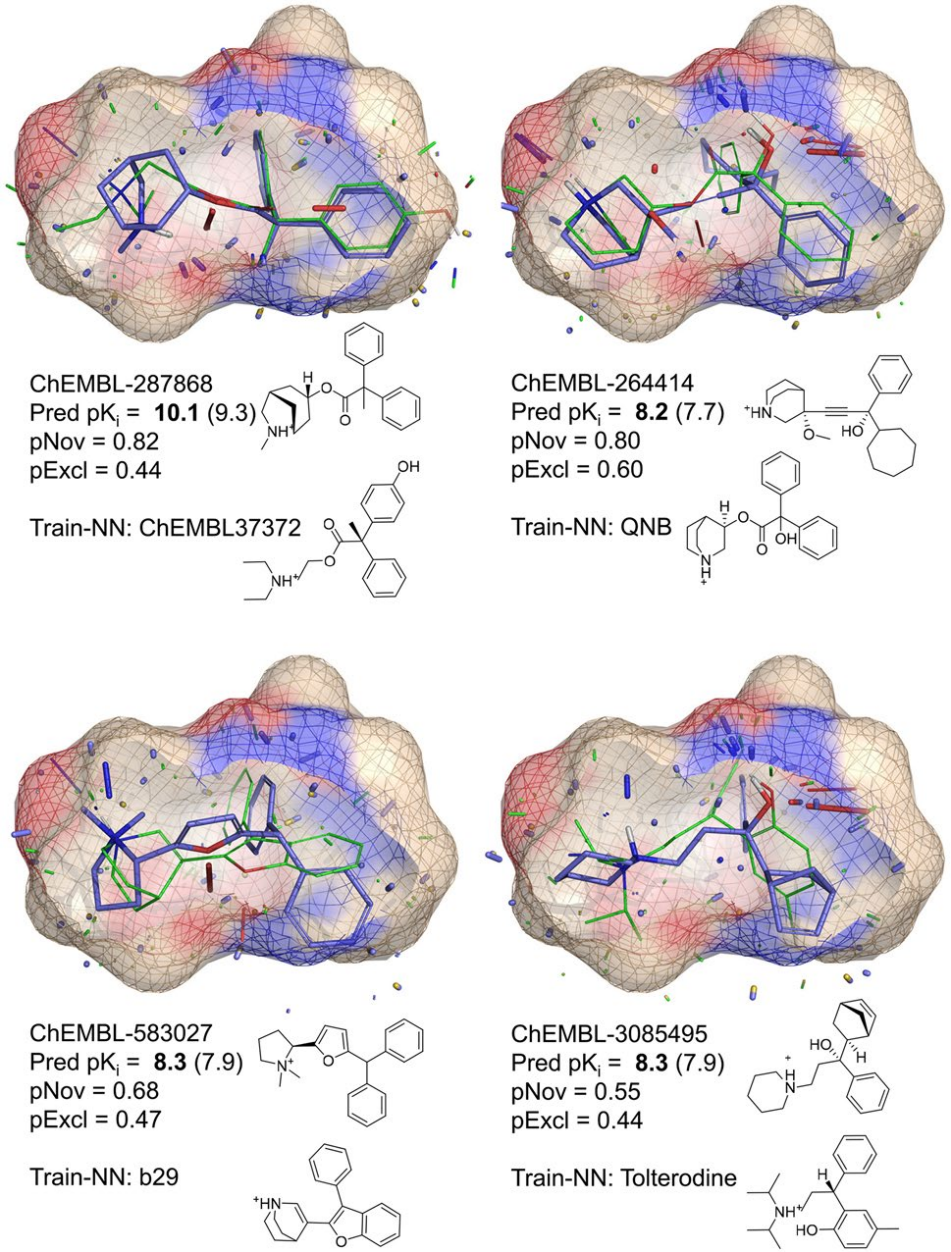
In-model QuanSA predictions of potent muscarinic antagonists of diverse scaffolds.

Figure 17 shows four examples of the predicted in-model potent ChEMBL molecules. The first compound (ChEMBL-287868) was the most potent, but least interesting, as it was an obvious analog of QNB, atropine, and related older antimuscarinics [56]. The second (ChEMBL-264414) was published in 2008 [57]. The muscarinic activity of alkyne linked quinuclidines was a surprise to researchers working toward novel bladder agents, and the selectivity profile suggested potential toward a COPD indication. This scaffold was different from any within the expanded training set for model refinement. The third compound, ChEMBL-583027, was reported in 2010 [58]. It was one of a group of pyrrolidinylfurans, of which two potent examples were identified by the unrefined model (ChEMBL compounds 571121 and 569760, predicted at 8.1 and 9.5, respectively, with actual pK$_i$ values of 8.3 and 8.0). The refined model, in addition to identifying ChEMBL-583027, also identified four other actives of this scaffold correctly.

The last compound (lower right) was identified through pharmacophoric modeling and virtual screening, reported in 2013 [59]. Of the 28 compounds from in that study, 12 were represented within the ChEMBL data set with reported $pK_i \ge 7.5$. The initial unrefined QuanSA model identified one-third of these (ChEMBL molecules 37372 and 517712 with experimental $pK_i> 9.0$, and 2377261 and 2377269, both with $pK_i$ very close to 8.0).

The refined model identified three more with activity predictions $\ge 7.5$. This included ChEMBL molecule 3085495 shown in Fig. 17. ChEMBL molecules 2106570 and 2377268 were predicted to have pK$_i$ values of 8.5 and 7.5 and had experimental activities of 8.8, and 7.5, respectively. The remaining five ChEMBL compounds (1231, 2377387, 1490, 1123, 2377267, all with experimental pK$_i \ge$ 7.5) were predicted with pK$_i =$ 6.5–6.9. The QuanSA model would have identified all of the potent compounds as in-model winners in a screen with the threshold set as pK$^{pred}_i \ge 6.5$, with an extremely low false positive rate (3/1000 ZINC decoys are identified by this procedure at this predicted activity threshold).

In all cases of predicted and confirmed active molecules, even for those with divergent scaffolds from all training examples, the pose predictions were consistent across multiple variants and were convincing in the light of the SAR available.

## Conclusions

We have described a new QSAR method, called QuanSA, which is a hybrid of sorts combining aspects of Compass and QMOD, two antecedent methods. QuanSA, however, is more sophisticated than either, being more physically realistic than Compass in every respect and allowing for modeling the subtleties of protein pocket variation in a pocket-field in ways that QMOD could not. The approach addresses the question of molecular pose completely automatically, including a means to select from among alternative molecular alignments. Ligand strain is modeled in a realistic manner, and calculations applying a pocket-field to new molecules make predictions of affinity, pose, and produce metrics that quantify the degree to which the data underlying a model support a prediction.

The approach was applied on sixteen separate data sets, eight of which allowed for direct comparison with the FEP method, four of which allowed for extensive testing on large ChEMBL data sets, and two of which place the results in context within the QSAR literature. The sets cover pharmaceutically relevant targets, including serine and aspartyl proteases, kinases, esterases, phosphatases, other enzymes, ligand-gated ion channels, and GPCRs. Predictive performance tracked with prediction difficulty. On the types of predictions suitable for FEP-style calculations, mean absolute error was roughly 0.5 pK$_d$ units (0.7 kcal/mol), with some particular cases approaching chemical accuracy. For challenging cases by conventional QSAR standards, typical errors on blind test molecules were about 0.7 log units (0.9 kcal/mol). In cases where it was possible to test QuanSA models on large sets of ChEMBL data, typical errors were higher, ranging from 0.9–1.5 log units (1.2–2.0 kcal/mol), but the models were able to identify potent and structurally novel molecules with high specificity and produced highly statistically significant rank orderings of potency.

In a detailed examination of an application scenario in which limited data were available (two series from a single discovery effort plus a handful of well-known contemporaneously available examples), a QuanSA model was able to induce an accurate model. This model matched, to a remarkable degree, the structure of the target’s binding pocket, published two decades after the lead optimization effort took place. Even difficult aspects of SAR were explained by the interplay between the induced pocket-field and the ligands under investigation.

The critical deviation of QuanSA from the large variety of QSAR methods is that it approaches building *causal* models. The models do not simply correlate with activity. They are physically realistic manifestations into which low-energy conformations of ligands must fit. However, this represents only a step in the right direction. Aspects of protein-ligand interactions that involve subtleties of entropy or kinetics are beyond what is currently envisioned within the scope of the method.

There are a number of areas for further improvement and validation. In particular, the speed of application to new molecules (typically tens of seconds) could be optimized further, especially toward large-scale evaluation of synthetic ideas. Most importantly, the multiple ligand alignment process could be optimized with respect to its objective function, which trades off mutual similarity, volume compactness, and ligand strain. Extensive optimization and validation of that procedure on a large number of targets is planned.
